# Search for a new heavy gauge-boson resonance decaying into a lepton and missing transverse momentum in 36 fb$^{-1}$ of *pp* collisions at $\sqrt{s} = 13$ TeV with the ATLAS experiment 

**DOI:** 10.1140/epjc/s10052-018-5877-y

**Published:** 2018-05-22

**Authors:** M. Aaboud, G. Aad, B. Abbott, O. Abdinov, B. Abeloos, S. H. Abidi, O. S. AbouZeid, N. L. Abraham, H. Abramowicz, H. Abreu, R. Abreu, Y. Abulaiti, B. S. Acharya, S. Adachi, L. Adamczyk, J. Adelman, M. Adersberger, T. Adye, A. A. Affolder, T. Agatonovic-Jovin, C. Agheorghiesei, J. A. Aguilar-Saavedra, S. P. Ahlen, F. Ahmadov, G. Aielli, S. Akatsuka, H. Akerstedt, T. P. A. Åkesson, E. Akilli, A. V. Akimov, G. L. Alberghi, J. Albert, P. Albicocco, M. J. Alconada Verzini, S. C. Alderweireldt, M. Aleksa, I. N. Aleksandrov, C. Alexa, G. Alexander, T. Alexopoulos, M. Alhroob, B. Ali, M. Aliev, G. Alimonti, J. Alison, S. P. Alkire, B. M. M. Allbrooke, B. W. Allen, P. P. Allport, A. Aloisio, A. Alonso, F. Alonso, C. Alpigiani, A. A. Alshehri, M. Alstaty, B. Alvarez Gonzalez, D. Álvarez Piqueras, M. G. Alviggi, B. T. Amadio, Y. Amaral Coutinho, C. Amelung, D. Amidei, S. P. Amor Dos Santos, A. Amorim, S. Amoroso, G. Amundsen, C. Anastopoulos, L. S. Ancu, N. Andari, T. Andeen, C. F. Anders, J. K. Anders, K. J. Anderson, A. Andreazza, V. Andrei, S. Angelidakis, I. Angelozzi, A. Angerami, A. V. Anisenkov, N. Anjos, A. Annovi, C. Antel, M. Antonelli, A. Antonov, D. J. Antrim, F. Anulli, M. Aoki, L. Aperio Bella, G. Arabidze, Y. Arai, J. P. Araque, V. Araujo Ferraz, A. T. H. Arce, R. E. Ardell, F. A. Arduh, J-F. Arguin, S. Argyropoulos, M. Arik, A. J. Armbruster, L. J. Armitage, O. Arnaez, H. Arnold, M. Arratia, O. Arslan, A. Artamonov, G. Artoni, S. Artz, S. Asai, N. Asbah, A. Ashkenazi, L. Asquith, K. Assamagan, R. Astalos, M. Atkinson, N. B. Atlay, K. Augsten, G. Avolio, B. Axen, M. K. Ayoub, G. Azuelos, A. E. Baas, M. J. Baca, H. Bachacou, K. Bachas, M. Backes, M. Backhaus, P. Bagnaia, M. Bahmani, H. Bahrasemani, J. T. Baines, M. Bajic, O. K. Baker, E. M. Baldin, P. Balek, F. Balli, W. K. Balunas, E. Banas, A. Bandyopadhyay, Sw. Banerjee, A. A. E. Bannoura, L. Barak, E. L. Barberio, D. Barberis, M. Barbero, T. Barillari, M-S Barisits, J. T. Barkeloo, T. Barklow, N. Barlow, S. L. Barnes, B. M. Barnett, R. M. Barnett, Z. Barnovska-Blenessy, A. Baroncelli, G. Barone, A. J. Barr, L. Barranco Navarro, F. Barreiro, J. Barreiro Guimarães da Costa, R. Bartoldus, A. E. Barton, P. Bartos, A. Basalaev, A. Bassalat, R. L. Bates, S. J. Batista, J. R. Batley, M. Battaglia, M. Bauce, F. Bauer, H. S. Bawa, J. B. Beacham, M. D. Beattie, T. Beau, P. H. Beauchemin, P. Bechtle, H. P. Beck, H. C. Beck, K. Becker, M. Becker, M. Beckingham, C. Becot, A. J. Beddall, A. Beddall, V. A. Bednyakov, M. Bedognetti, C. P. Bee, T. A. Beermann, M. Begalli, M. Begel, J. K. Behr, A. S. Bell, G. Bella, L. Bellagamba, A. Bellerive, M. Bellomo, K. Belotskiy, O. Beltramello, N. L. Belyaev, O. Benary, D. Benchekroun, M. Bender, K. Bendtz, N. Benekos, Y. Benhammou, E. Benhar Noccioli, J. Benitez, D. P. Benjamin, M. Benoit, J. R. Bensinger, S. Bentvelsen, L. Beresford, M. Beretta, D. Berge, E. Bergeaas Kuutmann, N. Berger, J. Beringer, S. Berlendis, N. R. Bernard, G. Bernardi, C. Bernius, F. U. Bernlochner, T. Berry, P. Berta, C. Bertella, G. Bertoli, F. Bertolucci, I. A. Bertram, C. Bertsche, D. Bertsche, G. J. Besjes, O. Bessidskaia Bylund, M. Bessner, N. Besson, C. Betancourt, A. Bethani, S. Bethke, A. J. Bevan, J. Beyer, R. M. Bianchi, O. Biebel, D. Biedermann, R. Bielski, K. Bierwagen, N. V. Biesuz, M. Biglietti, T. R. V. Billoud, H. Bilokon, M. Bindi, A. Bingul, C. Bini, S. Biondi, T. Bisanz, C. Bittrich, D. M. Bjergaard, C. W. Black, J. E. Black, K. M. Black, R. E. Blair, T. Blazek, I. Bloch, C. Blocker, A. Blue, W. Blum, U. Blumenschein, S. Blunier, G. J. Bobbink, V. S. Bobrovnikov, S. S. Bocchetta, A. Bocci, C. Bock, M. Boehler, D. Boerner, D. Bogavac, A. G. Bogdanchikov, C. Bohm, V. Boisvert, P. Bokan, T. Bold, A. S. Boldyrev, A. E. Bolz, M. Bomben, M. Bona, M. Boonekamp, A. Borisov, G. Borissov, J. Bortfeldt, D. Bortoletto, V. Bortolotto, D. Boscherini, M. Bosman, J. D. Bossio Sola, J. Boudreau, J. Bouffard, E. V. Bouhova-Thacker, D. Boumediene, C. Bourdarios, S. K. Boutle, A. Boveia, J. Boyd, I. R. Boyko, J. Bracinik, A. Brandt, G. Brandt, O. Brandt, U. Bratzler, B. Brau, J. E. Brau, W. D. Breaden Madden, K. Brendlinger, A. J. Brennan, L. Brenner, R. Brenner, S. Bressler, D. L. Briglin, T. M. Bristow, D. Britton, D. Britzger, F. M. Brochu, I. Brock, R. Brock, G. Brooijmans, T. Brooks, W. K. Brooks, J. Brosamer, E. Brost, J. H Broughton, P. A. Bruckman de Renstrom, D. Bruncko, A. Bruni, G. Bruni, L. S. Bruni, B H Brunt, M. Bruschi, N. Bruscino, P. Bryant, L. Bryngemark, T. Buanes, Q. Buat, P. Buchholz, A. G. Buckley, I. A. Budagov, F. Buehrer, M. K. Bugge, O. Bulekov, D. Bullock, T. J. Burch, S. Burdin, C. D. Burgard, A. M. Burger, B. Burghgrave, K. Burka, S. Burke, I. Burmeister, J. T. P. Burr, E. Busato, D. Büscher, V. Büscher, P. Bussey, J. M. Butler, C. M. Buttar, J. M. Butterworth, P. Butti, W. Buttinger, A. Buzatu, A. R. Buzykaev, S. Cabrera Urbán, D. Caforio, V. M. Cairo, O. Cakir, N. Calace, P. Calafiura, A. Calandri, G. Calderini, P. Calfayan, G. Callea, L. P. Caloba, S. Calvente Lopez, D. Calvet, S. Calvet, T. P. Calvet, R. Camacho Toro, S. Camarda, P. Camarri, D. Cameron, R. Caminal Armadans, C. Camincher, S. Campana, M. Campanelli, A. Camplani, A. Campoverde, V. Canale, M. Cano Bret, J. Cantero, T. Cao, M. D. M. Capeans Garrido, I. Caprini, M. Caprini, M. Capua, R. M. Carbone, R. Cardarelli, F. Cardillo, I. Carli, T. Carli, G. Carlino, B. T. Carlson, L. Carminati, R. M. D. Carney, S. Caron, E. Carquin, S. Carrá, G. D. Carrillo-Montoya, J. Carvalho, D. Casadei, M. P. Casado, M. Casolino, D. W. Casper, R. Castelijn, V. Castillo Gimenez, N. F. Castro, A. Catinaccio, J. R. Catmore, A. Cattai, J. Caudron, V. Cavaliere, E. Cavallaro, D. Cavalli, M. Cavalli-Sforza, V. Cavasinni, E. Celebi, F. Ceradini, L. Cerda Alberich, A. S. Cerqueira, A. Cerri, L. Cerrito, F. Cerutti, A. Cervelli, S. A. Cetin, A. Chafaq, D. Chakraborty, S. K. Chan, W. S. Chan, Y. L. Chan, P. Chang, J. D. Chapman, D. G. Charlton, C. C. Chau, C. A. Chavez Barajas, S. Che, S. Cheatham, A. Chegwidden, S. Chekanov, S. V. Chekulaev, G. A. Chelkov, M. A. Chelstowska, C. Chen, H. Chen, J. Chen, S. Chen, S. Chen, X. Chen, Y. Chen, H. C. Cheng, H. J. Cheng, A. Cheplakov, E. Cheremushkina, R. Cherkaoui El Moursli, E. Cheu, K. Cheung, L. Chevalier, V. Chiarella, G. Chiarelli, G. Chiodini, A. S. Chisholm, A. Chitan, Y. H. Chiu, M. V. Chizhov, K. Choi, A. R. Chomont, S. Chouridou, V. Christodoulou, D. Chromek-Burckhart, M. C. Chu, J. Chudoba, A. J. Chuinard, J. J. Chwastowski, L. Chytka, A. K. Ciftci, D. Cinca, V. Cindro, I. A. Cioara, C. Ciocca, A. Ciocio, F. Cirotto, Z. H. Citron, M. Citterio, M. Ciubancan, A. Clark, B. L. Clark, M. R. Clark, P. J. Clark, R. N. Clarke, C. Clement, Y. Coadou, M. Cobal, A. Coccaro, J. Cochran, L. Colasurdo, B. Cole, A. P. Colijn, J. Collot, T. Colombo, P. Conde Muiño, E. Coniavitis, S. H. Connell, I. A. Connelly, S. Constantinescu, G. Conti, F. Conventi, M. Cooke, A. M. Cooper-Sarkar, F. Cormier, K. J. R. Cormier, M. Corradi, F. Corriveau, A. Cortes-Gonzalez, G. Cortiana, G. Costa, M. J. Costa, D. Costanzo, G. Cottin, G. Cowan, B. E. Cox, K. Cranmer, S. J. Crawley, R. A. Creager, G. Cree, S. Crépé-Renaudin, F. Crescioli, W. A. Cribbs, M. Cristinziani, V. Croft, G. Crosetti, A. Cueto, T. Cuhadar Donszelmann, A. R. Cukierman, J. Cummings, M. Curatolo, J. Cúth, P. Czodrowski, G. D’amen, S. D’Auria, L. D’eramo, M. D’Onofrio, M. J. Da Cunha Sargedas De Sousa, C. Da Via, W. Dabrowski, T. Dado, T. Dai, O. Dale, F. Dallaire, C. Dallapiccola, M. Dam, J. R. Dandoy, M. F. Daneri, N. P. Dang, A. C. Daniells, N. S. Dann, M. Danninger, M. Dano Hoffmann, V. Dao, G. Darbo, S. Darmora, J. Dassoulas, A. Dattagupta, T. Daubney, W. Davey, C. David, T. Davidek, D. R. Davis, P. Davison, E. Dawe, I. Dawson, K. De, R. de Asmundis, A. De Benedetti, S. De Castro, S. De Cecco, N. De Groot, P. de Jong, H. De la Torre, F. De Lorenzi, A. De Maria, D. De Pedis, A. De Salvo, U. De Sanctis, A. De Santo, K. De Vasconcelos Corga, J. B. De Vivie De Regie, W. J. Dearnaley, R. Debbe, C. Debenedetti, D. V. Dedovich, N. Dehghanian, I. Deigaard, M. Del Gaudio, J. Del Peso, D. Delgove, F. Deliot, C. M. Delitzsch, A. Dell’Acqua, L. Dell’Asta, M. Dell’Orso, M. Della Pietra, D. della Volpe, M. Delmastro, C. Delporte, P. A. Delsart, D. A. DeMarco, S. Demers, M. Demichev, A. Demilly, S. P. Denisov, D. Denysiuk, D. Derendarz, J. E. Derkaoui, F. Derue, P. Dervan, K. Desch, C. Deterre, K. Dette, M. R. Devesa, P. O. Deviveiros, A. Dewhurst, S. Dhaliwal, F. A. Di Bello, A. Di Ciaccio, L. Di Ciaccio, W. K. Di Clemente, C. Di Donato, A. Di Girolamo, B. Di Girolamo, B. Di Micco, R. Di Nardo, K. F. Di Petrillo, A. Di Simone, R. Di Sipio, D. Di Valentino, C. Diaconu, M. Diamond, F. A. Dias, M. A. Diaz, E. B. Diehl, J. Dietrich, S. Díez Cornell, A. Dimitrievska, J. Dingfelder, P. Dita, S. Dita, F. Dittus, F. Djama, T. Djobava, J. I. Djuvsland, M. A. B. do Vale, D. Dobos, M. Dobre, C. Doglioni, J. Dolejsi, Z. Dolezal, M. Donadelli, S. Donati, P. Dondero, J. Donini, J. Dopke, A. Doria, M. T. Dova, A. T. Doyle, E. Drechsler, M. Dris, Y. Du, J. Duarte-Campderros, A. Dubreuil, E. Duchovni, G. Duckeck, A. Ducourthial, O. A. Ducu, D. Duda, A. Dudarev, A. Chr. Dudder, E. M. Duffield, L. Duflot, M. Dührssen, M. Dumancic, A. E. Dumitriu, A. K. Duncan, M. Dunford, H. Duran Yildiz, M. Düren, A. Durglishvili, D. Duschinger, B. Dutta, M. Dyndal, B. S. Dziedzic, C. Eckardt, K. M. Ecker, R. C. Edgar, T. Eifert, G. Eigen, K. Einsweiler, T. Ekelof, M. El Kacimi, R. El Kosseifi, V. Ellajosyula, M. Ellert, S. Elles, F. Ellinghaus, A. A. Elliot, N. Ellis, J. Elmsheuser, M. Elsing, D. Emeliyanov, Y. Enari, O. C. Endner, J. S. Ennis, J. Erdmann, A. Ereditato, M. Ernst, S. Errede, M. Escalier, C. Escobar, B. Esposito, O. Estrada Pastor, A. I. Etienvre, E. Etzion, H. Evans, A. Ezhilov, M. Ezzi, F. Fabbri, L. Fabbri, V. Fabiani, G. Facini, R. M. Fakhrutdinov, S. Falciano, R. J. Falla, J. Faltova, Y. Fang, M. Fanti, A. Farbin, A. Farilla, C. Farina, E. M. Farina, T. Farooque, S. Farrell, S. M. Farrington, P. Farthouat, F. Fassi, P. Fassnacht, D. Fassouliotis, M. Faucci Giannelli, A. Favareto, W. J. Fawcett, L. Fayard, O. L. Fedin, W. Fedorko, S. Feigl, L. Feligioni, C. Feng, E. J. Feng, H. Feng, M. J. Fenton, A. B. Fenyuk, L. Feremenga, P. Fernandez Martinez, S. Fernandez Perez, J. Ferrando, A. Ferrari, P. Ferrari, R. Ferrari, D. E. Ferreira de Lima, A. Ferrer, D. Ferrere, C. Ferretti, F. Fiedler, A. Filipčič, M. Filipuzzi, F. Filthaut, M. Fincke-Keeler, K. D. Finelli, M. C. N. Fiolhais, L. Fiorini, A. Fischer, C. Fischer, J. Fischer, W. C. Fisher, N. Flaschel, I. Fleck, P. Fleischmann, R. R. M. Fletcher, T. Flick, B. M. Flierl, L. R. Flores Castillo, M. J. Flowerdew, G. T. Forcolin, A. Formica, F. A. Förster, A. Forti, A. G. Foster, D. Fournier, H. Fox, S. Fracchia, P. Francavilla, M. Franchini, S. Franchino, D. Francis, L. Franconi, M. Franklin, M. Frate, M. Fraternali, D. Freeborn, S. M. Fressard-Batraneanu, B. Freund, D. Froidevaux, J. A. Frost, C. Fukunaga, T. Fusayasu, J. Fuster, C. Gabaldon, O. Gabizon, A. Gabrielli, A. Gabrielli, G. P. Gach, S. Gadatsch, S. Gadomski, G. Gagliardi, L. G. Gagnon, C. Galea, B. Galhardo, E. J. Gallas, B. J. Gallop, P. Gallus, G. Galster, K. K. Gan, S. Ganguly, Y. Gao, Y. S. Gao, F. M. Garay Walls, C. García, J. E. García Navarro, J. A. García Pascual, M. Garcia-Sciveres, R. W. Gardner, N. Garelli, V. Garonne, A. Gascon Bravo, K. Gasnikova, C. Gatti, A. Gaudiello, G. Gaudio, I. L. Gavrilenko, C. Gay, G. Gaycken, E. N. Gazis, C. N. P. Gee, J. Geisen, M. Geisen, M. P. Geisler, K. Gellerstedt, C. Gemme, M. H. Genest, C. Geng, S. Gentile, C. Gentsos, S. George, D. Gerbaudo, A. Gershon, P. Gessinger-Befurt, G. Geßner, S. Ghasemi, M. Ghneimat, B. Giacobbe, S. Giagu, N. Giangiacomi, P. Giannetti, S. M. Gibson, M. Gignac, M. Gilchriese, D. Gillberg, G. Gilles, D. M. Gingrich, N. Giokaris, M. P. Giordani, F. M. Giorgi, P. F. Giraud, P. Giromini, D. Giugni, F. Giuli, C. Giuliani, M. Giulini, B. K. Gjelsten, S. Gkaitatzis, I. Gkialas, E. L. Gkougkousis, P. Gkountoumis, L. K. Gladilin, C. Glasman, J. Glatzer, P. C. F. Glaysher, A. Glazov, M. Goblirsch-Kolb, J. Godlewski, S. Goldfarb, T. Golling, D. Golubkov, A. Gomes, R. Gonçalo, R. Goncalves Gama, J. Goncalves Pinto Firmino Da Costa, G. Gonella, L. Gonella, A. Gongadze, S. González de la Hoz, S. Gonzalez-Sevilla, L. Goossens, P. A. Gorbounov, H. A. Gordon, I. Gorelov, B. Gorini, E. Gorini, A. Gorišek, A. T. Goshaw, C. Gössling, M. I. Gostkin, C. A. Gottardo, C. R. Goudet, D. Goujdami, A. G. Goussiou, N. Govender, E. Gozani, L. Graber, I. Grabowska-Bold, P. O. J. Gradin, J. Gramling, E. Gramstad, S. Grancagnolo, V. Gratchev, P. M. Gravila, C. Gray, H. M. Gray, Z. D. Greenwood, C. Grefe, K. Gregersen, I. M. Gregor, P. Grenier, K. Grevtsov, J. Griffiths, A. A. Grillo, K. Grimm, S. Grinstein, Ph. Gris, J.-F. Grivaz, S. Groh, E. Gross, J. Grosse-Knetter, G. C. Grossi, Z. J. Grout, A. Grummer, L. Guan, W. Guan, J. Guenther, F. Guescini, D. Guest, O. Gueta, B. Gui, E. Guido, T. Guillemin, S. Guindon, U. Gul, C. Gumpert, J. Guo, W. Guo, Y. Guo, R. Gupta, S. Gupta, G. Gustavino, P. Gutierrez, N. G. Gutierrez Ortiz, C. Gutschow, C. Guyot, M. P. Guzik, C. Gwenlan, C. B. Gwilliam, A. Haas, C. Haber, H. K. Hadavand, N. Haddad, A. Hadef, S. Hageböck, M. Hagihara, H. Hakobyan, M. Haleem, J. Haley, G. Halladjian, G. D. Hallewell, K. Hamacher, P. Hamal, K. Hamano, A. Hamilton, G. N. Hamity, P. G. Hamnett, L. Han, S. Han, K. Hanagaki, K. Hanawa, M. Hance, B. Haney, P. Hanke, J. B. Hansen, J. D. Hansen, M. C. Hansen, P. H. Hansen, K. Hara, A. S. Hard, T. Harenberg, F. Hariri, S. Harkusha, R. D. Harrington, P. F. Harrison, N. M. Hartmann, M. Hasegawa, Y. Hasegawa, A. Hasib, S. Hassani, S. Haug, R. Hauser, L. Hauswald, L. B. Havener, M. Havranek, C. M. Hawkes, R. J. Hawkings, D. Hayakawa, D. Hayden, C. P. Hays, J. M. Hays, H. S. Hayward, S. J. Haywood, S. J. Head, T. Heck, V. Hedberg, L. Heelan, S. Heer, K. K. Heidegger, S. Heim, T. Heim, B. Heinemann, J. J. Heinrich, L. Heinrich, C. Heinz, J. Hejbal, L. Helary, A. Held, S. Hellman, C. Helsens, R. C. W. Henderson, Y. Heng, S. Henkelmann, A. M. Henriques Correia, S. Henrot-Versille, G. H. Herbert, H. Herde, V. Herget, Y. Hernández Jiménez, H. Herr, G. Herten, R. Hertenberger, L. Hervas, T. C. Herwig, G. G. Hesketh, N. P. Hessey, J. W. Hetherly, S. Higashino, E. Higón-Rodriguez, K. Hildebrand, E. Hill, J. C. Hill, K. H. Hiller, S. J. Hillier, M. Hils, I. Hinchliffe, M. Hirose, D. Hirschbuehl, B. Hiti, O. Hladik, X. Hoad, J. Hobbs, N. Hod, M. C. Hodgkinson, P. Hodgson, A. Hoecker, M. R. Hoeferkamp, F. Hoenig, D. Hohn, T. R. Holmes, M. Homann, S. Honda, T. Honda, T. M. Hong, B. H. Hooberman, W. H. Hopkins, Y. Horii, A. J. Horton, J-Y. Hostachy, S. Hou, A. Hoummada, J. Howarth, J. Hoya, M. Hrabovsky, J. Hrdinka, I. Hristova, J. Hrivnac, T. Hryn’ova, A. Hrynevich, P. J. Hsu, S.-C. Hsu, Q. Hu, S. Hu, Y. Huang, Z. Hubacek, F. Hubaut, F. Huegging, T. B. Huffman, E. W. Hughes, G. Hughes, M. Huhtinen, P. Huo, N. Huseynov, J. Huston, J. Huth, G. Iacobucci, G. Iakovidis, I. Ibragimov, L. Iconomidou-Fayard, Z. Idrissi, P. Iengo, O. Igonkina, T. Iizawa, Y. Ikegami, M. Ikeno, Y. Ilchenko, D. Iliadis, N. Ilic, G. Introzzi, P. Ioannou, M. Iodice, K. Iordanidou, V. Ippolito, M. F. Isacson, N. Ishijima, M. Ishino, M. Ishitsuka, C. Issever, S. Istin, F. Ito, J. M. Iturbe Ponce, R. Iuppa, H. Iwasaki, J. M. Izen, V. Izzo, S. Jabbar, P. Jackson, R. M. Jacobs, V. Jain, K. B. Jakobi, K. Jakobs, S. Jakobsen, T. Jakoubek, D. O. Jamin, D. K. Jana, R. Jansky, J. Janssen, M. Janus, P. A. Janus, G. Jarlskog, N. Javadov, T. Javůrek, M. Javurkova, F. Jeanneau, L. Jeanty, J. Jejelava, A. Jelinskas, P. Jenni, C. Jeske, S. Jézéquel, H. Ji, J. Jia, H. Jiang, Y. Jiang, Z. Jiang, S. Jiggins, J. Jimenez Pena, S. Jin, A. Jinaru, O. Jinnouchi, H. Jivan, P. Johansson, K. A. Johns, C. A. Johnson, W. J. Johnson, K. Jon-And, R. W. L. Jones, S. D. Jones, S. Jones, T. J. Jones, J. Jongmanns, P. M. Jorge, J. Jovicevic, X. Ju, A. Juste Rozas, M. K. Köhler, A. Kaczmarska, M. Kado, H. Kagan, M. Kagan, S. J. Kahn, T. Kaji, E. Kajomovitz, C. W. Kalderon, A. Kaluza, S. Kama, A. Kamenshchikov, N. Kanaya, L. Kanjir, V. A. Kantserov, J. Kanzaki, B. Kaplan, L. S. Kaplan, D. Kar, K. Karakostas, N. Karastathis, M. J. Kareem, E. Karentzos, S. N. Karpov, Z. M. Karpova, K. Karthik, V. Kartvelishvili, A. N. Karyukhin, K. Kasahara, L. Kashif, R. D. Kass, A. Kastanas, Y. Kataoka, C. Kato, A. Katre, J. Katzy, K. Kawade, K. Kawagoe, T. Kawamoto, G. Kawamura, E. F. Kay, V. F. Kazanin, R. Keeler, R. Kehoe, J. S. Keller, J. J. Kempster, J Kendrick, H. Keoshkerian, O. Kepka, B. P. Kerševan, S. Kersten, R. A. Keyes, M. Khader, F. Khalil-zada, A. Khanov, A. G. Kharlamov, T. Kharlamova, A. Khodinov, T. J. Khoo, V. Khovanskiy, E. Khramov, J. Khubua, S. Kido, C. R. Kilby, H. Y. Kim, S. H. Kim, Y. K. Kim, N. Kimura, O. M. Kind, B. T. King, D. Kirchmeier, J. Kirk, A. E. Kiryunin, T. Kishimoto, D. Kisielewska, V. Kitali, K. Kiuchi, O. Kivernyk, E. Kladiva, T. Klapdor-Kleingrothaus, M. H. Klein, M. Klein, U. Klein, K. Kleinknecht, P. Klimek, A. Klimentov, R. Klingenberg, T. Klingl, T. Klioutchnikova, E.-E. Kluge, P. Kluit, S. Kluth, E. Kneringer, E. B. F. G. Knoops, A. Knue, A. Kobayashi, D. Kobayashi, T. Kobayashi, M. Kobel, M. Kocian, P. Kodys, T. Koffas, E. Koffeman, N. M. Köhler, T. Koi, M. Kolb, I. Koletsou, A. A. Komar, Y. Komori, T. Kondo, N. Kondrashova, K. Köneke, A. C. König, T. Kono, R. Konoplich, N. Konstantinidis, R. Kopeliansky, S. Koperny, A. K. Kopp, K. Korcyl, K. Kordas, A. Korn, A. A. Korol, I. Korolkov, E. V. Korolkova, O. Kortner, S. Kortner, T. Kosek, V. V. Kostyukhin, A. Kotwal, A. Koulouris, A. Kourkoumeli-Charalampidi, C. Kourkoumelis, E. Kourlitis, V. Kouskoura, A. B. Kowalewska, R. Kowalewski, T. Z. Kowalski, C. Kozakai, W. Kozanecki, A. S. Kozhin, V. A. Kramarenko, G. Kramberger, D. Krasnopevtsev, M. W. Krasny, A. Krasznahorkay, D. Krauss, J. A. Kremer, J. Kretzschmar, K. Kreutzfeldt, P. Krieger, K. Krizka, K. Kroeninger, H. Kroha, J. Kroll, J. Kroll, J. Kroseberg, J. Krstic, U. Kruchonak, H. Krüger, N. Krumnack, M. C. Kruse, T. Kubota, H. Kucuk, S. Kuday, J. T. Kuechler, S. Kuehn, A. Kugel, F. Kuger, T. Kuhl, V. Kukhtin, R. Kukla, Y. Kulchitsky, S. Kuleshov, Y. P. Kulinich, M. Kuna, T. Kunigo, A. Kupco, T. Kupfer, O. Kuprash, H. Kurashige, L. L. Kurchaninov, Y. A. Kurochkin, M. G. Kurth, V. Kus, E. S. Kuwertz, M. Kuze, J. Kvita, T. Kwan, D. Kyriazopoulos, A. La Rosa, J. L. La Rosa Navarro, L. La Rotonda, F. La Ruffa, C. Lacasta, F. Lacava, J. Lacey, H. Lacker, D. Lacour, E. Ladygin, R. Lafaye, B. Laforge, T. Lagouri, S. Lai, S. Lammers, W. Lampl, E. Lançon, U. Landgraf, M. P. J. Landon, M. C. Lanfermann, V. S. Lang, J. C. Lange, R. J. Langenberg, A. J. Lankford, F. Lanni, K. Lantzsch, A. Lanza, A. Lapertosa, S. Laplace, J. F. Laporte, T. Lari, F. Lasagni Manghi, M. Lassnig, P. Laurelli, W. Lavrijsen, A. T. Law, P. Laycock, T. Lazovich, M. Lazzaroni, B. Le, O. Le Dortz, E. Le Guirriec, E. P. Le Quilleuc, M. LeBlanc, T. LeCompte, F. Ledroit-Guillon, C. A. Lee, G. R. Lee, S. C. Lee, L. Lee, B. Lefebvre, G. Lefebvre, M. Lefebvre, F. Legger, C. Leggett, G. Lehmann Miotto, X. Lei, W. A. Leight, M. A. L. Leite, R. Leitner, D. Lellouch, B. Lemmer, K. J. C. Leney, T. Lenz, B. Lenzi, R. Leone, S. Leone, C. Leonidopoulos, G. Lerner, C. Leroy, A. A. J. Lesage, C. G. Lester, M. Levchenko, J. Levêque, D. Levin, L. J. Levinson, M. Levy, D. Lewis, B. Li, Changqiao Li, H. Li, L. Li, Q. Li, S. Li, X. Li, Y. Li, Z. Liang, B. Liberti, A. Liblong, K. Lie, J. Liebal, W. Liebig, A. Limosani, S. C. Lin, T. H. Lin, B. E. Lindquist, A. E. Lionti, E. Lipeles, A. Lipniacka, M. Lisovyi, T. M. Liss, A. Lister, A. M. Litke, B. Liu, H. Liu, H. Liu, J. K. K. Liu, J. Liu, J. B. Liu, K. Liu, L. Liu, M. Liu, Y. L. Liu, Y. Liu, M. Livan, A. Lleres, J. Llorente Merino, S. L. Lloyd, C. Y. Lo, F. Lo Sterzo, E. M. Lobodzinska, P. Loch, F. K. Loebinger, A. Loesle, K. M. Loew, A. Loginov, T. Lohse, K. Lohwasser, M. Lokajicek, B. A. Long, J. D. Long, R. E. Long, L. Longo, K. A. Looper, J. A. Lopez, D. Lopez Mateos, I. Lopez Paz, A. Lopez Solis, J. Lorenz, N. Lorenzo Martinez, M. Losada, P. J. Lösel, X. Lou, A. Lounis, J. Love, P. A. Love, H. Lu, N. Lu, Y. J. Lu, H. J. Lubatti, C. Luci, A. Lucotte, C. Luedtke, F. Luehring, W. Lukas, L. Luminari, O. Lundberg, B. Lund-Jensen, M. S. Lutz, P. M. Luzi, D. Lynn, R. Lysak, E. Lytken, F. Lyu, V. Lyubushkin, H. Ma, L. L. Ma, Y. Ma, G. Maccarrone, A. Macchiolo, C. M. Macdonald, B. Maček, J. Machado Miguens, D. Madaffari, R. Madar, W. F. Mader, A. Madsen, J. Maeda, S. Maeland, T. Maeno, A. S. Maevskiy, V. Magerl, J. Mahlstedt, C. Maiani, C. Maidantchik, A. A. Maier, T. Maier, A. Maio, O. Majersky, S. Majewski, Y. Makida, N. Makovec, B. Malaescu, Pa. Malecki, V. P. Maleev, F. Malek, U. Mallik, D. Malon, C. Malone, S. Maltezos, S. Malyukov, J. Mamuzic, G. Mancini, I. Mandić, J. Maneira, L. Manhaes de Andrade Filho, J. Manjarres Ramos, K. H. Mankinen, A. Mann, A. Manousos, B. Mansoulie, J. D. Mansour, R. Mantifel, M. Mantoani, S. Manzoni, L. Mapelli, G. Marceca, L. March, L. Marchese, G. Marchiori, M. Marcisovsky, M. Marjanovic, D. E. Marley, F. Marroquim, S. P. Marsden, Z. Marshall, M. U. F Martensson, S. Marti-Garcia, C. B. Martin, T. A. Martin, V. J. Martin, B. Martin dit Latour, M. Martinez, V. I. Martinez Outschoorn, S. Martin-Haugh, V. S. Martoiu, A. C. Martyniuk, A. Marzin, L. Masetti, T. Mashimo, R. Mashinistov, J. Masik, A. L. Maslennikov, L. Massa, P. Mastrandrea, A. Mastroberardino, T. Masubuchi, P. Mättig, J. Maurer, S. J. Maxfield, D. A. Maximov, R. Mazini, I. Maznas, S. M. Mazza, N. C. Mc Fadden, G. Mc Goldrick, S. P. Mc Kee, A. McCarn, R. L. McCarthy, T. G. McCarthy, L. I. McClymont, E. F. McDonald, J. A. Mcfayden, G. Mchedlidze, S. J. McMahon, P. C. McNamara, R. A. McPherson, S. Meehan, T. J. Megy, S. Mehlhase, A. Mehta, T. Meideck, K. Meier, B. Meirose, D. Melini, B. R. Mellado Garcia, J. D. Mellenthin, M. Melo, F. Meloni, A. Melzer, S. B. Menary, L. Meng, X. T. Meng, A. Mengarelli, S. Menke, E. Meoni, S. Mergelmeyer, P. Mermod, L. Merola, C. Meroni, F. S. Merritt, A. Messina, J. Metcalfe, A. S. Mete, C. Meyer, J-P. Meyer, J. Meyer, H. Meyer Zu Theenhausen, F. Miano, R. P. Middleton, S. Miglioranzi, L. Mijović, G. Mikenberg, M. Mikestikova, M. Mikuž, M. Milesi, A. Milic, D. W. Miller, C. Mills, A. Milov, D. A. Milstead, A. A. Minaenko, Y. Minami, I. A. Minashvili, A. I. Mincer, B. Mindur, M. Mineev, Y. Minegishi, Y. Ming, L. M. Mir, K. P. Mistry, T. Mitani, J. Mitrevski, V. A. Mitsou, A. Miucci, P. S. Miyagawa, A. Mizukami, J. U. Mjörnmark, T. Mkrtchyan, M. Mlynarikova, T. Moa, K. Mochizuki, P. Mogg, S. Mohapatra, S. Molander, R. Moles-Valls, R. Monden, M. C. Mondragon, K. Mönig, J. Monk, E. Monnier, A. Montalbano, J. Montejo Berlingen, F. Monticelli, S. Monzani, R. W. Moore, N. Morange, D. Moreno, M. Moreno Llácer, P. Morettini, S. Morgenstern, D. Mori, T. Mori, M. Morii, M. Morinaga, V. Morisbak, A. K. Morley, G. Mornacchi, J. D. Morris, L. Morvaj, P. Moschovakos, M. Mosidze, H. J. Moss, J. Moss, K. Motohashi, R. Mount, E. Mountricha, E. J. W. Moyse, S. Muanza, F. Mueller, J. Mueller, R. S. P. Mueller, D. Muenstermann, P. Mullen, G. A. Mullier, F. J. Munoz Sanchez, W. J. Murray, H. Musheghyan, M. Muškinja, A. G. Myagkov, M. Myska, B. P. Nachman, O. Nackenhorst, K. Nagai, R. Nagai, K. Nagano, Y. Nagasaka, K. Nagata, M. Nagel, E. Nagy, A. M. Nairz, Y. Nakahama, K. Nakamura, T. Nakamura, I. Nakano, R. F. Naranjo Garcia, R. Narayan, D. I. Narrias Villar, I. Naryshkin, T. Naumann, G. Navarro, R. Nayyar, H. A. Neal, P. Yu. Nechaeva, T. J. Neep, A. Negri, M. Negrini, S. Nektarijevic, C. Nellist, A. Nelson, M. E. Nelson, S. Nemecek, P. Nemethy, M. Nessi, M. S. Neubauer, M. Neumann, P. R. Newman, T. Y. Ng, T. Nguyen Manh, R. B. Nickerson, R. Nicolaidou, J. Nielsen, V. Nikolaenko, I. Nikolic-Audit, K. Nikolopoulos, J. K. Nilsen, P. Nilsson, Y. Ninomiya, A. Nisati, N. Nishu, R. Nisius, I. Nitsche, T. Nitta, T. Nobe, Y. Noguchi, M. Nomachi, I. Nomidis, M. A. Nomura, T. Nooney, M. Nordberg, N. Norjoharuddeen, O. Novgorodova, S. Nowak, M. Nozaki, L. Nozka, K. Ntekas, E. Nurse, F. Nuti, K. O’connor, D. C. O’Neil, A. A. O’Rourke, V. O’Shea, F. G. Oakham, H. Oberlack, T. Obermann, J. Ocariz, A. Ochi, I. Ochoa, J. P. Ochoa-Ricoux, S. Oda, S. Odaka, A. Oh, S. H. Oh, C. C. Ohm, H. Ohman, H. Oide, H. Okawa, Y. Okumura, T. Okuyama, A. Olariu, L. F. Oleiro Seabra, S. A. Olivares Pino, D. Oliveira Damazio, A. Olszewski, J. Olszowska, A. Onofre, K. Onogi, P. U. E. Onyisi, H. Oppen, M. J. Oreglia, Y. Oren, D. Orestano, N. Orlando, R. S. Orr, B. Osculati, R. Ospanov, G. Otero y Garzon, H. Otono, M. Ouchrif, F. Ould-Saada, A. Ouraou, K. P. Oussoren, Q. Ouyang, M. Owen, R. E. Owen, V. E. Ozcan, N. Ozturk, K. Pachal, A. Pacheco Pages, L. Pacheco Rodriguez, C. Padilla Aranda, S. Pagan Griso, M. Paganini, F. Paige, G. Palacino, S. Palazzo, S. Palestini, M. Palka, D. Pallin, E. St. Panagiotopoulou, I. Panagoulias, C. E. Pandini, J. G. Panduro Vazquez, P. Pani, S. Panitkin, D. Pantea, L. Paolozzi, Th. D. Papadopoulou, K. Papageorgiou, A. Paramonov, D. Paredes Hernandez, A. J. Parker, M. A. Parker, K. A. Parker, F. Parodi, J. A. Parsons, U. Parzefall, V. R. Pascuzzi, J. M. Pasner, E. Pasqualucci, S. Passaggio, Fr. Pastore, S. Pataraia, J. R. Pater, T. Pauly, B. Pearson, S. Pedraza Lopez, R. Pedro, S. V. Peleganchuk, O. Penc, C. Peng, H. Peng, J. Penwell, B. S. Peralva, M. M. Perego, D. V. Perepelitsa, F. Peri, L. Perini, H. Pernegger, S. Perrella, R. Peschke, V. D. Peshekhonov, K. Peters, R. F. Y. Peters, B. A. Petersen, T. C. Petersen, E. Petit, A. Petridis, C. Petridou, P. Petroff, E. Petrolo, M. Petrov, F. Petrucci, N. E. Pettersson, A. Peyaud, R. Pezoa, F. H. Phillips, P. W. Phillips, G. Piacquadio, E. Pianori, A. Picazio, E. Piccaro, M. A. Pickering, R. Piegaia, J. E. Pilcher, A. D. Pilkington, A. W. J. Pin, M. Pinamonti, J. L. Pinfold, H. Pirumov, M. Pitt, L. Plazak, M.-A. Pleier, V. Pleskot, E. Plotnikova, D. Pluth, P. Podberezko, R. Poettgen, R. Poggi, L. Poggioli, D. Pohl, G. Polesello, A. Poley, A. Policicchio, R. Polifka, A. Polini, C. S. Pollard, V. Polychronakos, K. Pommès, D. Ponomarenko, L. Pontecorvo, G. A. Popeneciu, A. Poppleton, S. Pospisil, K. Potamianos, I. N. Potrap, C. J. Potter, G. Poulard, T. Poulsen, J. Poveda, M. E. Pozo Astigarraga, P. Pralavorio, A. Pranko, S. Prell, D. Price, M. Primavera, S. Prince, N. Proklova, K. Prokofiev, F. Prokoshin, S. Protopopescu, J. Proudfoot, M. Przybycien, A. Puri, P. Puzo, J. Qian, G. Qin, Y. Qin, A. Quadt, M. Queitsch-Maitland, D. Quilty, S. Raddum, V. Radeka, V. Radescu, S. K. Radhakrishnan, P. Radloff, P. Rados, F. Ragusa, G. Rahal, J. A. Raine, S. Rajagopalan, C. Rangel-Smith, T. Rashid, S. Raspopov, M. G. Ratti, D. M. Rauch, F. Rauscher, S. Rave, I. Ravinovich, J. H. Rawling, M. Raymond, A. L. Read, N. P. Readioff, M. Reale, D. M. Rebuzzi, A. Redelbach, G. Redlinger, R. Reece, R. G. Reed, K. Reeves, L. Rehnisch, J. Reichert, A. Reiss, C. Rembser, H. Ren, M. Rescigno, S. Resconi, E. D. Resseguie, S. Rettie, E. Reynolds, O. L. Rezanova, P. Reznicek, R. Rezvani, R. Richter, S. Richter, E. Richter-Was, O. Ricken, M. Ridel, P. Rieck, C. J. Riegel, J. Rieger, O. Rifki, M. Rijssenbeek, A. Rimoldi, M. Rimoldi, L. Rinaldi, G. Ripellino, B. Ristić, E. Ritsch, I. Riu, F. Rizatdinova, E. Rizvi, C. Rizzi, R. T. Roberts, S. H. Robertson, A. Robichaud-Veronneau, D. Robinson, J. E. M. Robinson, A. Robson, E. Rocco, C. Roda, Y. Rodina, S. Rodriguez Bosca, A. Rodriguez Perez, D. Rodriguez Rodriguez, S. Roe, C. S. Rogan, O. Røhne, J. Roloff, A. Romaniouk, M. Romano, S. M. Romano Saez, E. Romero Adam, N. Rompotis, M. Ronzani, L. Roos, S. Rosati, K. Rosbach, P. Rose, N.-A. Rosien, E. Rossi, L. P. Rossi, J. H. N. Rosten, R. Rosten, M. Rotaru, J. Rothberg, D. Rousseau, A. Rozanov, Y. Rozen, X. Ruan, F. Rubbo, F. Rühr, A. Ruiz-Martinez, Z. Rurikova, N. A. Rusakovich, H. L. Russell, J. P. Rutherfoord, N. Ruthmann, Y. F. Ryabov, M. Rybar, G. Rybkin, S. Ryu, A. Ryzhov, G. F. Rzehorz, A. F. Saavedra, G. Sabato, S. Sacerdoti, H. F-W. Sadrozinski, R. Sadykov, F. Safai Tehrani, P. Saha, M. Sahinsoy, M. Saimpert, M. Saito, T. Saito, H. Sakamoto, Y. Sakurai, G. Salamanna, J. E. Salazar Loyola, D. Salek, P. H. Sales De Bruin, D. Salihagic, A. Salnikov, J. Salt, D. Salvatore, F. Salvatore, A. Salvucci, A. Salzburger, D. Sammel, D. Sampsonidis, D. Sampsonidou, J. Sánchez, V. Sanchez Martinez, A. Sanchez Pineda, H. Sandaker, R. L. Sandbach, C. O. Sander, M. Sandhoff, C. Sandoval, D. P. C. Sankey, M. Sannino, Y. Sano, A. Sansoni, C. Santoni, H. Santos, I. Santoyo Castillo, A. Sapronov, J. G. Saraiva, B. Sarrazin, O. Sasaki, K. Sato, E. Sauvan, G. Savage, P. Savard, N. Savic, C. Sawyer, L. Sawyer, J. Saxon, C. Sbarra, A. Sbrizzi, T. Scanlon, D. A. Scannicchio, M. Scarcella, J. Schaarschmidt, P. Schacht, B. M. Schachtner, D. Schaefer, L. Schaefer, R. Schaefer, J. Schaeffer, S. Schaepe, S. Schaetzel, U. Schäfer, A. C. Schaffer, D. Schaile, R. D. Schamberger, V. A. Schegelsky, D. Scheirich, M. Schernau, C. Schiavi, S. Schier, L. K. Schildgen, C. Schillo, M. Schioppa, S. Schlenker, K. R. Schmidt-Sommerfeld, K. Schmieden, C. Schmitt, S. Schmitt, S. Schmitz, U. Schnoor, L. Schoeffel, A. Schoening, B. D. Schoenrock, E. Schopf, M. Schott, J. F. P. Schouwenberg, J. Schovancova, S. Schramm, N. Schuh, A. Schulte, M. J. Schultens, H.-C. Schultz-Coulon, H. Schulz, M. Schumacher, B. A. Schumm, Ph. Schune, A. Schwartzman, T. A. Schwarz, H. Schweiger, Ph. Schwemling, R. Schwienhorst, J. Schwindling, A. Sciandra, G. Sciolla, M. Scornajenghi, F. Scuri, F. Scutti, J. Searcy, P. Seema, S. C. Seidel, A. Seiden, J. M. Seixas, G. Sekhniaidze, K. Sekhon, S. J. Sekula, N. Semprini-Cesari, S. Senkin, C. Serfon, L. Serin, L. Serkin, M. Sessa, R. Seuster, H. Severini, T. Sfiligoj, F. Sforza, A. Sfyrla, E. Shabalina, N. W. Shaikh, L. Y. Shan, R. Shang, J. T. Shank, M. Shapiro, P. B. Shatalov, K. Shaw, S. M. Shaw, A. Shcherbakova, C. Y. Shehu, Y. Shen, N. Sherafati, P. Sherwood, L. Shi, S. Shimizu, C. O. Shimmin, M. Shimojima, I. P. J. Shipsey, S. Shirabe, M. Shiyakova, J. Shlomi, A. Shmeleva, D. Shoaleh Saadi, M. J. Shochet, S. Shojaii, D. R. Shope, S. Shrestha, E. Shulga, M. A. Shupe, P. Sicho, A. M. Sickles, P. E. Sidebo, E. Sideras Haddad, O. Sidiropoulou, A. Sidoti, F. Siegert, Dj. Sijacki, J. Silva, S. B. Silverstein, V. Simak, Lj. Simic, S. Simion, E. Simioni, B. Simmons, M. Simon, P. Sinervo, N. B. Sinev, M. Sioli, G. Siragusa, I. Siral, S. Yu. Sivoklokov, J. Sjölin, M. B. Skinner, P. Skubic, M. Slater, T. Slavicek, M. Slawinska, K. Sliwa, R. Slovak, V. Smakhtin, B. H. Smart, J. Smiesko, N. Smirnov, S. Yu. Smirnov, Y. Smirnov, L. N. Smirnova, O. Smirnova, J. W. Smith, M. N. K. Smith, R. W. Smith, M. Smizanska, K. Smolek, A. A. Snesarev, I. M. Snyder, S. Snyder, R. Sobie, F. Socher, A. Soffer, A. Søgaard, D. A. Soh, G. Sokhrannyi, C. A. Solans Sanchez, M. Solar, E. Yu. Soldatov, U. Soldevila, A. A. Solodkov, A. Soloshenko, O. V. Solovyanov, V. Solovyev, P. Sommer, H. Son, A. Sopczak, D. Sosa, C. L. Sotiropoulou, R. Soualah, A. M. Soukharev, D. South, B. C. Sowden, S. Spagnolo, M. Spalla, M. Spangenberg, F. Spanò, D. Sperlich, F. Spettel, T. M. Spieker, R. Spighi, G. Spigo, L. A. Spiller, M. Spousta, R. D. St. Denis, A. Stabile, R. Stamen, S. Stamm, E. Stanecka, R. W. Stanek, C. Stanescu, M. M. Stanitzki, B. S. Stapf, S. Stapnes, E. A. Starchenko, G. H. Stark, J. Stark, S. H Stark, P. Staroba, P. Starovoitov, S. Stärz, R. Staszewski, P. Steinberg, B. Stelzer, H. J. Stelzer, O. Stelzer-Chilton, H. Stenzel, G. A. Stewart, M. C. Stockton, M. Stoebe, G. Stoicea, P. Stolte, S. Stonjek, A. R. Stradling, A. Straessner, M. E. Stramaglia, J. Strandberg, S. Strandberg, M. Strauss, P. Strizenec, R. Ströhmer, D. M. Strom, R. Stroynowski, A. Strubig, S. A. Stucci, B. Stugu, N. A. Styles, D. Su, J. Su, S. Suchek, Y. Sugaya, M. Suk, V. V. Sulin, D M S Sultan, S. Sultansoy, T. Sumida, S. Sun, X. Sun, K. Suruliz, C. J. E. Suster, M. R. Sutton, S. Suzuki, M. Svatos, M. Swiatlowski, S. P. Swift, I. Sykora, T. Sykora, D. Ta, K. Tackmann, J. Taenzer, A. Taffard, R. Tafirout, N. Taiblum, H. Takai, R. Takashima, E. H. Takasugi, T. Takeshita, Y. Takubo, M. Talby, A. A. Talyshev, J. Tanaka, M. Tanaka, R. Tanaka, S. Tanaka, R. Tanioka, B. B. Tannenwald, S. Tapia Araya, S. Tapprogge, S. Tarem, G. F. Tartarelli, P. Tas, M. Tasevsky, T. Tashiro, E. Tassi, A. Tavares Delgado, Y. Tayalati, A. C. Taylor, G. N. Taylor, P. T. E. Taylor, W. Taylor, P. Teixeira-Dias, D. Temple, H. Ten Kate, P. K. Teng, J. J. Teoh, F. Tepel, S. Terada, K. Terashi, J. Terron, S. Terzo, M. Testa, R. J. Teuscher, T. Theveneaux-Pelzer, F. Thiele, J. P. Thomas, J. Thomas-Wilsker, P. D. Thompson, A. S. Thompson, L. A. Thomsen, E. Thomson, M. J. Tibbetts, R. E. Ticse Torres, V. O. Tikhomirov, Yu. A. Tikhonov, S. Timoshenko, P. Tipton, S. Tisserant, K. Todome, S. Todorova-Nova, S. Todt, J. Tojo, S. Tokár, K. Tokushuku, E. Tolley, L. Tomlinson, M. Tomoto, L. Tompkins, K. Toms, B. Tong, P. Tornambe, E. Torrence, H. Torres, E. Torró Pastor, J. Toth, F. Touchard, D. R. Tovey, C. J. Treado, T. Trefzger, F. Tresoldi, A. Tricoli, I. M. Trigger, S. Trincaz-Duvoid, M. F. Tripiana, W. Trischuk, B. Trocmé, A. Trofymov, C. Troncon, M. Trottier-McDonald, M. Trovatelli, L. Truong, M. Trzebinski, A. Trzupek, K. W. Tsang, J. C-L. Tseng, P. V. Tsiareshka, G. Tsipolitis, N. Tsirintanis, S. Tsiskaridze, V. Tsiskaridze, E. G. Tskhadadze, K. M. Tsui, I. I. Tsukerman, V. Tsulaia, S. Tsuno, D. Tsybychev, Y. Tu, A. Tudorache, V. Tudorache, T. T. Tulbure, A. N. Tuna, S. A. Tupputi, S. Turchikhin, D. Turgeman, I. Turk Cakir, R. Turra, P. M. Tuts, G. Ucchielli, I. Ueda, M. Ughetto, F. Ukegawa, G. Unal, A. Undrus, G. Unel, F. C. Ungaro, Y. Unno, C. Unverdorben, J. Urban, P. Urquijo, P. Urrejola, G. Usai, J. Usui, L. Vacavant, V. Vacek, B. Vachon, K. O. H. Vadla, A. Vaidya, C. Valderanis, E. Valdes Santurio, S. Valentinetti, A. Valero, L. Valéry, S. Valkar, A. Vallier, J. A. Valls Ferrer, W. Van Den Wollenberg, H. van der Graaf, P. van Gemmeren, J. Van Nieuwkoop, I. van Vulpen, M. C. van Woerden, M. Vanadia, W. Vandelli, A. Vaniachine, P. Vankov, G. Vardanyan, R. Vari, E. W. Varnes, C. Varni, T. Varol, D. Varouchas, A. Vartapetian, K. E. Varvell, J. G. Vasquez, G. A. Vasquez, F. Vazeille, T. Vazquez Schroeder, J. Veatch, V. Veeraraghavan, L. M. Veloce, F. Veloso, S. Veneziano, A. Ventura, M. Venturi, N. Venturi, A. Venturini, V. Vercesi, M. Verducci, W. Verkerke, A. T. Vermeulen, J. C. Vermeulen, M. C. Vetterli, N. Viaux Maira, O. Viazlo, I. Vichou, T. Vickey, O. E. Vickey Boeriu, G. H. A. Viehhauser, S. Viel, L. Vigani, M. Villa, M. Villaplana Perez, E. Vilucchi, M. G. Vincter, V. B. Vinogradov, A. Vishwakarma, C. Vittori, I. Vivarelli, S. Vlachos, M. Vogel, P. Vokac, G. Volpi, H. von der Schmitt, E. von Toerne, V. Vorobel, K. Vorobev, M. Vos, R. Voss, J. H. Vossebeld, N. Vranjes, M. Vranjes Milosavljevic, V. Vrba, M. Vreeswijk, R. Vuillermet, I. Vukotic, P. Wagner, W. Wagner, J. Wagner-Kuhr, H. Wahlberg, S. Wahrmund, J. Wakabayashi, J. Walder, R. Walker, W. Walkowiak, V. Wallangen, C. Wang, C. Wang, F. Wang, H. Wang, H. Wang, J. Wang, J. Wang, Q. Wang, R. Wang, S. M. Wang, T. Wang, W. Wang, W. Wang, Z. Wang, C. Wanotayaroj, A. Warburton, C. P. Ward, D. R. Wardrope, A. Washbrook, P. M. Watkins, A. T. Watson, M. F. Watson, G. Watts, S. Watts, B. M. Waugh, A. F. Webb, S. Webb, M. S. Weber, S. W. Weber, S. A. Weber, J. S. Webster, A. R. Weidberg, B. Weinert, J. Weingarten, M. Weirich, C. Weiser, H. Weits, P. S. Wells, T. Wenaus, T. Wengler, S. Wenig, N. Wermes, M. D. Werner, P. Werner, M. Wessels, K. Whalen, N. L. Whallon, A. M. Wharton, A. S. White, A. White, M. J. White, R. White, D. Whiteson, B. W. Whitmore, F. J. Wickens, W. Wiedenmann, M. Wielers, C. Wiglesworth, L. A. M. Wiik-Fuchs, A. Wildauer, F. Wilk, H. G. Wilkens, H. H. Williams, S. Williams, C. Willis, S. Willocq, J. A. Wilson, I. Wingerter-Seez, E. Winkels, F. Winklmeier, O. J. Winston, B. T. Winter, M. Wittgen, M. Wobisch, T. M. H. Wolf, R. Wolff, M. W. Wolter, H. Wolters, V. W. S. Wong, S. D. Worm, B. K. Wosiek, J. Wotschack, K. W. Wozniak, M. Wu, S. L. Wu, X. Wu, Y. Wu, T. R. Wyatt, B. M. Wynne, S. Xella, Z. Xi, L. Xia, D. Xu, L. Xu, T. Xu, B. Yabsley, S. Yacoob, D. Yamaguchi, Y. Yamaguchi, A. Yamamoto, S. Yamamoto, T. Yamanaka, M. Yamatani, K. Yamauchi, Y. Yamazaki, Z. Yan, H. Yang, H. Yang, Y. Yang, Z. Yang, W-M. Yao, Y. C. Yap, Y. Yasu, E. Yatsenko, K. H. Yau Wong, J. Ye, S. Ye, I. Yeletskikh, E. Yigitbasi, E. Yildirim, K. Yorita, K. Yoshihara, C. Young, C. J. S. Young, J. Yu, J. Yu, S. P. Y. Yuen, I. Yusuff, B. Zabinski, G. Zacharis, R. Zaidan, A. M. Zaitsev, N. Zakharchuk, J. Zalieckas, A. Zaman, S. Zambito, D. Zanzi, C. Zeitnitz, G. Zemaityte, A. Zemla, J. C. Zeng, Q. Zeng, O. Zenin, T. Ženiš, D. Zerwas, D. Zhang, F. Zhang, G. Zhang, H. Zhang, J. Zhang, L. Zhang, L. Zhang, M. Zhang, P. Zhang, R. Zhang, R. Zhang, X. Zhang, Y. Zhang, Z. Zhang, X. Zhao, Y. Zhao, Z. Zhao, A. Zhemchugov, B. Zhou, C. Zhou, L. Zhou, M. Zhou, M. Zhou, N. Zhou, C. G. Zhu, H. Zhu, J. Zhu, Y. Zhu, X. Zhuang, K. Zhukov, A. Zibell, D. Zieminska, N. I. Zimine, C. Zimmermann, S. Zimmermann, Z. Zinonos, M. Zinser, M. Ziolkowski, L. Živković, G. Zobernig, A. Zoccoli, R. Zou, M. zur Nedden, L. Zwalinski, ATLAS Collaboration

**Affiliations:** 1 Department of Physics, University of Adelaide, Adelaide, Australia; 2 Physics Department, SUNY Albany, Albany, NY USA; 3 Department of Physics, University of Alberta, Edmonton, AB Canada; 4 Department of Physics, Ankara University, Ankara, Turkey; 5 Istanbul Aydin University, Istanbul, Turkey; 6 Division of Physics, TOBB University of Economics and Technology, Ankara, Turkey; 7 LAPP, CNRS/IN2P3 and Université Savoie Mont Blanc, Annecy-le-Vieux, France; 8 High Energy Physics Division, Argonne National Laboratory, Argonne, IL USA; 9 Department of Physics, University of Arizona, Tucson, AZ USA; 10 Department of Physics, The University of Texas at Arlington, Arlington, TX USA; 11 Physics Department, National and Kapodistrian University of Athens, Athens, Greece; 12 Physics Department, National Technical University of Athens, Zografou, Greece; 13 Department of Physics, The University of Texas at Austin, Austin, TX USA; 14 Institute of Physics, Azerbaijan Academy of Sciences, Baku, Azerbaijan; 15 Institut de Física d’Altes Energies (IFAE), The Barcelona Institute of Science and Technology, Barcelona, Spain; 16 Institute of Physics, University of Belgrade, Belgrade, Serbia; 17 Department for Physics and Technology, University of Bergen, Bergen, Norway; 18 Physics Division, Lawrence Berkeley National Laboratory, University of California, Berkeley, CA USA; 19 Department of Physics, Humboldt University, Berlin, Germany; 20 Albert Einstein Center for Fundamental Physics, Laboratory for High Energy Physics, University of Bern, Bern, Switzerland; 21 School of Physics and Astronomy, University of Birmingham, Birmingham, UK; 22 Department of Physics, Bogazici University, Istanbul, Turkey; 23 Department of Physics Engineering, Gaziantep University, Gaziantep, Turkey; 24 Faculty of Engineering and Natural Sciences, Istanbul Bilgi University, Istanbul, Turkey; 25 Faculty of Engineering and Natural Sciences, Bahcesehir University, Istanbul, Turkey; 26 Centro de Investigaciones, Universidad Antonio Narino, Bogotá, Colombia; 27 INFN Sezione di Bologna, Bologna, Italy; 28 Dipartimento di Fisica e Astronomia, Università di Bologna, Bologna, Italy; 29 Physikalisches Institut, University of Bonn, Bonn, Germany; 30 Department of Physics, Boston University, Boston, MA USA; 31 Department of Physics, Brandeis University, Waltham, MA USA; 32 Universidade Federal do Rio De Janeiro COPPE/EE/IF, Rio de Janeiro, Brazil; 33 Electrical Circuits Department, Federal University of Juiz de Fora (UFJF), Juiz de Fora, Brazil; 34 Federal University of Sao Joao del Rei (UFSJ), Sao Joao del Rei, Brazil; 35 Instituto de Fisica, Universidade de Sao Paulo, São Paulo, Brazil; 36 Physics Department, Brookhaven National Laboratory, Upton, NY USA; 37 Transilvania University of Brasov, Brasov, Romania; 38 Horia Hulubei National Institute of Physics and Nuclear Engineering, Bucharest, Romania; 39 Department of Physics, Alexandru Ioan Cuza University of Iasi, Iasi, Romania; 40 Physics Department, National Institute for Research and Development of Isotopic and Molecular Technologies, Cluj-Napoca, Romania; 41 University Politehnica Bucharest, Bucharest, Romania; 42 West University in Timisoara, Timisoara, Romania; 43 Departamento de Física, Universidad de Buenos Aires, Buenos Aires, Argentina; 44 Cavendish Laboratory, University of Cambridge, Cambridge, UK; 45 Department of Physics, Carleton University, Ottawa, ON Canada; 46 CERN, Geneva, Switzerland; 47 Enrico Fermi Institute, University of Chicago, Chicago, IL USA; 48 Departamento de Física, Pontificia Universidad Católica de Chile, Santiago, Chile; 49 Departamento de Física, Universidad Técnica Federico Santa María, Valparaiso, Chile; 50 Institute of High Energy Physics, Chinese Academy of Sciences, Beijing, China; 51 Department of Physics, Nanjing University, Nanjing, Jiangsu China; 52 Physics Department, Tsinghua University, Beijing, 100084 China; 53 Department of Modern Physics and State Key Laboratory of Particle Detection and Electronics, University of Science and Technology of China, Hefei, Anhui China; 54 School of Physics, Shandong University, Shandong, China; 55 Department of Physics and Astronomy, Key Laboratory for Particle Physics, Astrophysics and Cosmology, Ministry of Education, Shanghai Key Laboratory for Particle Physics and Cosmology, Shanghai Jiao Tong University, Shanghai (also at PKU-CHEP), Shanghai, China; 56 Université Clermont Auvergne, CNRS/IN2P3, LPC, Clermont-Ferrand, France; 57 Nevis Laboratory, Columbia University, Irvington, NY USA; 58 Niels Bohr Institute, University of Copenhagen, Copenhagen, Denmark; 59 INFN Gruppo Collegato di Cosenza, Laboratori Nazionali di Frascati, Frascati, Italy; 60 Dipartimento di Fisica, Università della Calabria, Rende, Italy; 61 Faculty of Physics and Applied Computer Science, AGH University of Science and Technology, Kraków, Poland; 62 Marian Smoluchowski Institute of Physics, Jagiellonian University, Kraków, Poland; 63 Institute of Nuclear Physics, Polish Academy of Sciences, Kraków, Poland; 64 Physics Department, Southern Methodist University, Dallas, TX USA; 65 Physics Department, University of Texas at Dallas, Richardson, TX USA; 66 DESY, Hamburg and Zeuthen, Germany; 67 Lehrstuhl für Experimentelle Physik IV, Technische Universität Dortmund, Dortmund, Germany; 68 Institut für Kern- und Teilchenphysik, Technische Universität Dresden, Dresden, Germany; 69 Department of Physics, Duke University, Durham, NC USA; 70 SUPA-School of Physics and Astronomy, University of Edinburgh, Edinburgh, UK; 71 INFN e Laboratori Nazionali di Frascati, Frascati, Italy; 72 Fakultät für Mathematik und Physik, Albert-Ludwigs-Universität, Freiburg, Germany; 73 Departement de Physique Nucleaire et Corpusculaire, Université de Genève, Geneva, Switzerland; 74 INFN Sezione di Genova, Genoa, Italy; 75 Dipartimento di Fisica, Università di Genova, Genoa, Italy; 76 E. Andronikashvili Institute of Physics, Iv. Javakhishvili Tbilisi State University, Tbilisi, Georgia; 77 High Energy Physics Institute, Tbilisi State University, Tbilisi, Georgia; 78 II Physikalisches Institut, Justus-Liebig-Universität Giessen, Giessen, Germany; 79 SUPA-School of Physics and Astronomy, University of Glasgow, Glasgow, UK; 80 II Physikalisches Institut, Georg-August-Universität, Göttingen, Germany; 81 Laboratoire de Physique Subatomique et de Cosmologie, Université Grenoble-Alpes, CNRS/IN2P3, Grenoble, France; 82 Laboratory for Particle Physics and Cosmology, Harvard University, Cambridge, MA USA; 83 Kirchhoff-Institut für Physik, Ruprecht-Karls-Universität Heidelberg, Heidelberg, Germany; 84 Physikalisches Institut, Ruprecht-Karls-Universität Heidelberg, Heidelberg, Germany; 85 Faculty of Applied Information Science, Hiroshima Institute of Technology, Hiroshima, Japan; 86 Department of Physics, The Chinese University of Hong Kong, Shatin, NT Hong Kong; 87 Department of Physics, The University of Hong Kong, Hong Kong, China; 88 Department of Physics, Institute for Advanced Study, The Hong Kong University of Science and Technology, Clear Water Bay, Kowloon, Hong Kong, China; 89 Department of Physics, National Tsing Hua University, Taiwan, Taiwan; 90 Department of Physics, Indiana University, Bloomington, IN USA; 91 Institut für Astro- und Teilchenphysik, Leopold-Franzens-Universität, Innsbruck, Austria; 92 University of Iowa, Iowa City, IA USA; 93 Department of Physics and Astronomy, Iowa State University, Ames, IA USA; 94 Joint Institute for Nuclear Research, JINR Dubna, Dubna, Russia; 95 KEK, High Energy Accelerator Research Organization, Tsukuba, Japan; 96 Graduate School of Science, Kobe University, Kobe, Japan; 97 Faculty of Science, Kyoto University, Kyoto, Japan; 98 Kyoto University of Education, Kyoto, Japan; 99 Research Center for Advanced Particle Physics and Department of Physics, Kyushu University, Fukuoka, Japan; 100 Instituto de Física La Plata, Universidad Nacional de La Plata and CONICET, La Plata, Argentina; 101 Physics Department, Lancaster University, Lancaster, UK; 102 INFN Sezione di Lecce, Lecce, Italy; 103 Dipartimento di Matematica e Fisica, Università del Salento, Lecce, Italy; 104 Oliver Lodge Laboratory, University of Liverpool, Liverpool, UK; 105 Department of Experimental Particle Physics, Jožef Stefan Institute and Department of Physics, University of Ljubljana, Ljubljana, Slovenia; 106 School of Physics and Astronomy, Queen Mary University of London, London, UK; 107 Department of Physics, Royal Holloway University of London, Surrey, UK; 108 Department of Physics and Astronomy, University College London, London, UK; 109 Louisiana Tech University, Ruston, LA USA; 110 Laboratoire de Physique Nucléaire et de Hautes Energies, UPMC and Université Paris-Diderot and CNRS/IN2P3, Paris, France; 111 Fysiska institutionen, Lunds universitet, Lund, Sweden; 112 Departamento de Fisica Teorica C-15, Universidad Autonoma de Madrid, Madrid, Spain; 113 Institut für Physik, Universität Mainz, Mainz, Germany; 114 School of Physics and Astronomy, University of Manchester, Manchester, UK; 115 CPPM, Aix-Marseille Université and CNRS/IN2P3, Marseille, France; 116 Department of Physics, University of Massachusetts, Amherst, MA USA; 117 Department of Physics, McGill University, Montreal, QC Canada; 118 School of Physics, University of Melbourne, Victoria, Australia; 119 Department of Physics, The University of Michigan, Ann Arbor, MI USA; 120 Department of Physics and Astronomy, Michigan State University, East Lansing, MI USA; 121 INFN Sezione di Milano, Milan, Italy; 122 Dipartimento di Fisica, Università di Milano, Milan, Italy; 123 B.I. Stepanov Institute of Physics, National Academy of Sciences of Belarus, Minsk, Republic of Belarus; 124 Research Institute for Nuclear Problems of Byelorussian State University, Minsk, Republic of Belarus; 125 Group of Particle Physics, University of Montreal, Montreal, QC Canada; 126 P.N. Lebedev Physical Institute of the Russian Academy of Sciences, Moscow, Russia; 127 Institute for Theoretical and Experimental Physics (ITEP), Moscow, Russia; 128 National Research Nuclear University MEPhI, Moscow, Russia; 129 D.V. Skobeltsyn Institute of Nuclear Physics, M.V. Lomonosov Moscow State University, Moscow, Russia; 130 Fakultät für Physik, Ludwig-Maximilians-Universität München, Munich, Germany; 131 Max-Planck-Institut für Physik (Werner-Heisenberg-Institut), Munich, Germany; 132 Nagasaki Institute of Applied Science, Nagasaki, Japan; 133 Graduate School of Science and Kobayashi-Maskawa Institute, Nagoya University, Nagoya, Japan; 134 INFN Sezione di Napoli, Naples, Italy; 135 Dipartimento di Fisica, Università di Napoli, Naples, Italy; 136 Department of Physics and Astronomy, University of New Mexico, Albuquerque, NM USA; 137 Institute for Mathematics, Astrophysics and Particle Physics, Radboud University Nijmegen/Nikhef, Nijmegen, The Netherlands; 138 Nikhef National Institute for Subatomic Physics, University of Amsterdam, Amsterdam, The Netherlands; 139 Department of Physics, Northern Illinois University, DeKalb, IL USA; 140 Budker Institute of Nuclear Physics, SB RAS, Novosibirsk, Russia; 141 Department of Physics, New York University, New York, NY USA; 142 Ohio State University, Columbus, OH USA; 143 Faculty of Science, Okayama University, Okayama, Japan; 144 Homer L. Dodge Department of Physics and Astronomy, University of Oklahoma, Norman, OK USA; 145 Department of Physics, Oklahoma State University, Stillwater, OK USA; 146 Palacký University, RCPTM, Olomouc, Czech Republic; 147 Center for High Energy Physics, University of Oregon, Eugene, OR USA; 148 LAL, Univ. Paris-Sud, CNRS/IN2P3, Université Paris-Saclay, Orsay, France; 149 Graduate School of Science, Osaka University, Osaka, Japan; 150 Department of Physics, University of Oslo, Oslo, Norway; 151 Department of Physics, Oxford University, Oxford, UK; 152 INFN Sezione di Pavia, Pavia, Italy; 153 Dipartimento di Fisica, Università di Pavia, Pavia, Italy; 154 Department of Physics, University of Pennsylvania, Philadelphia, PA USA; 155 National Research Centre “Kurchatov Institute” B.P. Konstantinov Petersburg Nuclear Physics Institute, St. Petersburg, Russia; 156 INFN Sezione di Pisa, Pisa, Italy; 157 Dipartimento di Fisica E. Fermi, Università di Pisa, Pisa, Italy; 158 Department of Physics and Astronomy, University of Pittsburgh, Pittsburgh, PA USA; 159 Laboratório de Instrumentação e Física Experimental de Partículas-LIP, Lisbon, Portugal; 160 Faculdade de Ciências, Universidade de Lisboa, Lisbon, Portugal; 161 Department of Physics, University of Coimbra, Coimbra, Portugal; 162 Centro de Física Nuclear da Universidade de Lisboa, Lisbon, Portugal; 163 Departamento de Fisica, Universidade do Minho, Braga, Portugal; 164 Departamento de Fisica Teorica y del Cosmos and CAFPE, Universidad de Granada, Granada, Spain; 165 Dep Fisica and CEFITEC of Faculdade de Ciencias e Tecnologia, Universidade Nova de Lisboa, Caparica, Portugal; 166 Institute of Physics, Academy of Sciences of the Czech Republic, Prague, Czech Republic; 167 Czech Technical University in Prague, Prague, Czech Republic; 168 Faculty of Mathematics and Physics, Charles University, Prague, Czech Republic; 169 State Research Center Institute for High Energy Physics (Protvino), NRC KI, Protvino, Russia; 170 Particle Physics Department, Rutherford Appleton Laboratory, Didcot, UK; 171 INFN Sezione di Roma, Rome, Italy; 172 Dipartimento di Fisica, Sapienza Università di Roma, Rome, Italy; 173 INFN Sezione di Roma Tor Vergata, Rome, Italy; 174 Dipartimento di Fisica, Università di Roma Tor Vergata, Rome, Italy; 175 INFN Sezione di Roma Tre, Rome, Italy; 176 Dipartimento di Matematica e Fisica, Università Roma Tre, Rome, Italy; 177 Faculté des Sciences Ain Chock, Réseau Universitaire de Physique des Hautes Energies-Université Hassan II, Casablanca, Morocco; 178 Centre National de l’Energie des Sciences Techniques Nucleaires, Rabat, Morocco; 179 Faculté des Sciences Semlalia, Université Cadi Ayyad, LPHEA-Marrakech, Marrakech, Morocco; 180 Faculté des Sciences, Université Mohamed Premier and LPTPM, Oujda, Morocco; 181 Faculté des Sciences, Université Mohammed V, Rabat, Morocco; 182 DSM/IRFU (Institut de Recherches sur les Lois Fondamentales de l’Univers), CEA Saclay (Commissariat à l’Energie Atomique et aux Energies Alternatives), Gif-sur-Yvette, France; 183 Santa Cruz Institute for Particle Physics, University of California Santa Cruz, Santa Cruz, CA USA; 184 Department of Physics, University of Washington, Seattle, WA USA; 185 Department of Physics and Astronomy, University of Sheffield, Sheffield, UK; 186 Department of Physics, Shinshu University, Nagano, Japan; 187 Department Physik, Universität Siegen, Siegen, Germany; 188 Department of Physics, Simon Fraser University, Burnaby, BC Canada; 189 SLAC National Accelerator Laboratory, Stanford, CA USA; 190 Faculty of Mathematics, Physics and Informatics, Comenius University, Bratislava, Slovak Republic; 191 Department of Subnuclear Physics, Institute of Experimental Physics of the Slovak Academy of Sciences, Kosice, Slovak Republic; 192 Department of Physics, University of Cape Town, Cape Town, South Africa; 193 Department of Physics, University of Johannesburg, Johannesburg, South Africa; 194 School of Physics, University of the Witwatersrand, Johannesburg, South Africa; 195 Department of Physics, Stockholm University, Stockholm, Sweden; 196 The Oskar Klein Centre, Stockholm, Sweden; 197 Physics Department, Royal Institute of Technology, Stockholm, Sweden; 198 Departments of Physics and Astronomy and Chemistry, Stony Brook University, Stony Brook, NY USA; 199 Department of Physics and Astronomy, University of Sussex, Brighton, UK; 200 School of Physics, University of Sydney, Sydney, Australia; 201 Institute of Physics, Academia Sinica, Taipei, Taiwan; 202 Department of Physics, Technion: Israel Institute of Technology, Haifa, Israel; 203 Raymond and Beverly Sackler School of Physics and Astronomy, Tel Aviv University, Tel Aviv, Israel; 204 Department of Physics, Aristotle University of Thessaloniki, Thessaloníki, Greece; 205 International Center for Elementary Particle Physics and Department of Physics, The University of Tokyo, Tokyo, Japan; 206 Graduate School of Science and Technology, Tokyo Metropolitan University, Tokyo, Japan; 207 Department of Physics, Tokyo Institute of Technology, Tokyo, Japan; 208 Tomsk State University, Tomsk, Russia; 209 Department of Physics, University of Toronto, Toronto, ON Canada; 210 INFN-TIFPA, Trento, Italy; 211 University of Trento, Trento, Italy; 212 TRIUMF, Vancouver, BC Canada; 213 Department of Physics and Astronomy, York University, Toronto, ON Canada; 214 Faculty of Pure and Applied Sciences, and Center for Integrated Research in Fundamental Science and Engineering, University of Tsukuba, Tsukuba, Japan; 215 Department of Physics and Astronomy, Tufts University, Medford, MA USA; 216 Department of Physics and Astronomy, University of California Irvine, Irvine, CA USA; 217 INFN Gruppo Collegato di Udine, Sezione di Trieste, Udine, Italy; 218 ICTP, Trieste, Italy; 219 Dipartimento di Chimica, Fisica e Ambiente, Università di Udine, Udine, Italy; 220 Department of Physics and Astronomy, University of Uppsala, Uppsala, Sweden; 221 Department of Physics, University of Illinois, Urbana, IL USA; 222 Instituto de Fisica Corpuscular (IFIC), Centro Mixto Universidad de Valencia-CSIC, Valencia, Spain; 223 Department of Physics, University of British Columbia, Vancouver, BC Canada; 224 Department of Physics and Astronomy, University of Victoria, Victoria, BC Canada; 225 Department of Physics, University of Warwick, Coventry, UK; 226 Waseda University, Tokyo, Japan; 227 Department of Particle Physics, The Weizmann Institute of Science, Rehovot, Israel; 228 Department of Physics, University of Wisconsin, Madison, WI USA; 229 Fakultät für Physik und Astronomie, Julius-Maximilians-Universität, Würzburg, Germany; 230 Fakultät für Mathematik und Naturwissenschaften, Fachgruppe Physik, Bergische Universität Wuppertal, Wuppertal, Germany; 231 Department of Physics, Yale University, New Haven, CT USA; 232 Yerevan Physics Institute, Yerevan, Armenia; 233 Centre de Calcul de l’Institut National de Physique Nucléaire et de Physique des Particules (IN2P3), Villeurbanne, France; 234 Academia Sinica Grid Computing, Institute of Physics, Academia Sinica, Taipei, Taiwan; 235 CERN, 1211 Geneva 23, Switzerland

## Abstract

The results of a search for new heavy $W^\prime$ bosons decaying to an electron or muon and a neutrino using proton–proton collision data at a centre-of-mass energy of $\sqrt{s}~=~13$ TeV are presented. The dataset was collected in 2015 and 2016 by the ATLAS experiment at the Large Hadron Collider and corresponds to an integrated luminosity of 36.1 $\text{ fb }^{-1}$. As no excess of events above the Standard Model prediction is observed, the results are used to set upper limits on the $W^\prime$ boson cross-section times branching ratio to an electron or muon and a neutrino as a function of the $W^\prime$ mass. Assuming a $W^\prime$ boson with the same couplings as the Standard Model *W* boson, $W^\prime$ masses below 5.1 TeV are excluded at the 95% confidence level.

## Introduction

Extensions to the Standard Model (SM) may include heavy gauge bosons that could be discovered at the Large Hadron Collider (LHC) [1]. For example, heavy gauge bosons are predicted in left-right symmetric models [2, 3] or in the little Higgs model [4]. Conceptually, these particles are heavier versions of the SM *W* and *Z* bosons and are generically referred to as $W^\prime$ and $Z^\prime$ bosons. The Sequential Standard Model (SSM) [5] posits a $W'_{\mathrm {SSM}}$ boson with couplings to fermions that are identical to those of the SM *W* boson. This model represents a good benchmark as the results can be interpreted in the context of other models of new physics, and is useful for comparing the sensitivity of different experiments.

This paper presents a search for a $W^\prime$ boson conducted in the $W^\prime \rightarrow \ell \nu$ channel. In the following, the term *lepton* ($\ell$) is used to refer to an electron or a muon. The analysis uses events with a high transverse momentum^1^ ($p_{\text {T}}$) lepton and significant missing transverse momentum $E_{\text {T}}^{\text {miss}}$, that is used to infer the presence of the neutrino in the event as it escapes direct detection. It is based on 36.1 $\text{ fb }^{-1}$ of *pp* collision data collected with the ATLAS detector in 2015 and 2016 at a centre-of-mass energy of $\sqrt{s} = 13$ TeV. The results are interpreted in the context of the SSM. The signal discriminant is the transverse mass, which is defined as $\smash [b]{m_{\mathrm T}} = \sqrt{\smash [b]{2p_{\text {T}} E_{\text {T}}^{\text {miss}} (1-\cos \phi _{\ell \nu })}}$, where $\smash [b]{\phi _{\ell \nu }}$ is the azimuthal angle between the directions of the lepton $p_{\text {T}}$ and the $E_{\text {T}}^{\text {miss}}$ in the transverse plane.

The most stringent limits on the mass of a $W'_{\mathrm {SSM}}$ boson to date come from the searches in the $W^\prime \rightarrow e \nu$ and $W^\prime \rightarrow \mu \nu$ channels by the ATLAS and CMS collaborations using data taken at $\sqrt{s} = 13$ TeV in 2015. The ATLAS analysis was based on data corresponding to an integrated luminosity of 3.2 $\text{ fb }^{-1}$ and sets a 95% confidence level (CL) lower limit on the $W'_{\mathrm {SSM}}$ mass of 4.07 TeV [6]. The CMS Collaboration used 2.4 $\text{ fb }^{-1}$ of data and excludes $W'_{\mathrm {SSM}}$ masses below 4.1 TeV at 95% CL [7]. The sensitivity of the search presented here is significantly improved compared to these earlier searches due to the larger dataset.

## ATLAS detector

The ATLAS experiment [8] at the LHC is a multipurpose particle detector with a forward-backward symmetric cylindrical geometry and a near $4 \pi$ coverage in solid angle. It consists of an inner detector (ID) for tracking surrounded by a thin superconducting solenoid providing a 2 T axial magnetic field, electromagnetic (EM) and hadronic calorimeters, and a muon spectrometer (MS). The ID covers the pseudorapidity range $|\eta |<2.5$. It consists of a silicon pixel detector including an additional inner layer located at a radius of 3.2 cm since 2015 [9], followed by silicon microstrip and transition radiation tracking detectors. Lead/liquid-argon (LAr) sampling calorimeters provide EM energy measurements with high granularity. A hadronic (steel/scintillator-tile) calorimeter covers the central pseudorapidity range ($|\eta |<1.7$). The end-cap and forward regions are instrumented with LAr calorimeters for both the EM and hadronic energy measurements up to $|\eta |=4.9$. The muon spectrometer surrounds the calorimeters and is based on three large air-core toroidal superconducting magnets with eight coils each. The field integral experienced by tracks in the toroidal field ranges between 2.0 and 6.0 T m for most pseudorapidities. The MS includes a system of precision tracking chambers, over $|\eta | < 2.7$, and fast detectors for triggering, over $|\eta | < 2.4$. A two-level trigger system is used to select events [10]. The first-level trigger is implemented in hardware and uses a subset of the detector information. This is followed by a software-based trigger system that reduces the accepted event rate to less than 1 kHz.

## Analysis strategy and modelling of signal and background processes

A $W^\prime$ signal would appear as an excess of events above the SM background at high $m_{{\mathrm {T}}}$. The SM background mainly arises from processes with at least one prompt final-state lepton, with the largest source being the charged-current Drell–Yan (DY) *W* boson production, where the *W* boson decays into an electron or muon and a neutrino. The second largest source is top-quark pair ($t\bar{t}$) and single-top-quark production, denoted in the following as “top-quark background”. Other non-negligible contributions are from the neutral-current DY ($Z/\gamma ^*$) process, diboson production, as well as from events in which one final-state jet or photon satisfies the lepton selection criteria. This last component of the background, referred to in the following as the multijet background, receives contributions from multijet, heavy-flavour quark and $\gamma$ + jet production. The multijet background is determined using a data-driven method, while the other backgrounds are modelled by Monte Carlo (MC) simulations.

The backgrounds from $W \rightarrow \ell \nu$, $Z/\gamma ^*\rightarrow \ell \ell$, $W \rightarrow \tau \nu$, and $Z/\gamma ^*\rightarrow \tau \tau$ were simulated using the Powheg-Box v2 [11] matrix-element calculation up to next-to-leading order (NLO) in perturbative quantum chromodynamics (pQCD), interfaced to the Pythia 8.186 [12] parton shower model and using the CT10 parton distribution function (PDF) set [13]. The final-state photon radiation (QED FSR) was modelled by the Photos [14] MC simulation. The samples are normalised as a function of the boson invariant mass to a next-to-next-to-leading order (NNLO) pQCD calculation using the numerical programme VRAP  which is based on Ref. [15] and the CT14NNLO PDF set [16]. Compared to the NLO prediction using CT10, the NNLO prediction using CT14 gives a higher cross-section by about 5% at a boson invariant mass of 1 TeV and 10% at 5 TeV. In addition to the modelling of QED FSR, a fixed-order electroweak (EW) correction to NLO is calculated as a function of the boson mass with the Mcsanc [17, 18] event generator at leading order (LO) in pQCD. This correction is added to the NNLO QCD cross-section prediction in the so-called additive approach (see Sect. 6.2) because of a lack of calculations of mixed QCD and EW terms, and lowers the predicted cross-section by an increasing amount as function of the mass, reaching about 10% at 1 TeV and 20% at 5 TeV. The $W \rightarrow \ell \nu$ and $Z/\gamma ^*\rightarrow \ell \ell$ events were simulated as multiple samples covering different ranges of the boson invariant mass. This ensures that a large number of MC events is available across the entire $m_{{\mathrm {T}}}$ region probed in this analysis.

The background from $t\bar{t}$ production was generated using Powheg-Box v2, with parton showering and hadronisation modelled by Pythia 6.428 [19], using the CT10 PDF set. The $t\bar{t}$ cross-section is normalised to $\sigma _{t\bar{t}}= 832$ pb as calculated with the Top++2.0 program at NNLO in pQCD, including soft-gluon resummation to next-to-next-to-leading logarithmic accuracy (see Ref. [20] and references therein). The top-quark mass is set to 172.5 GeV. The single-top-quark production in the *Wt* channel and EW *t*-channel was simulated using the same event generators and PDF sets as for the $t\bar{t}$ process, with the exception that the Powheg-Box v1 program was used for producing events in the *t*-channel. Diboson events were simulated with the Sherpa 2.1.1 [21] event generator using the CT10 PDF set. As the simulated top-quark and diboson samples are statistically limited at large $m_{{\mathrm {T}}}$, the expected number of events from each of these backgrounds is extrapolated into the high-$m_{{\mathrm {T}}}$ region. This is achieved by fitting the lower part of the $m_{{\mathrm {T}}}$ distributions to functions of the form $F (x) = a \, x^{b+c \log x}$ and $F (x) = d/ (x + e)^g$, where $x = m_{\mathrm T}/\sqrt{s}$, and using the fitted function to predict the background at higher $m_{{\mathrm {T}}}$. Various fit ranges are used, which typically start between 140 and 360 GeV and extend up to 500–1300 GeV. The fits with the best $\chi ^2/{\text {d.o.f.}}$ are used for the extrapolation and the results of these fits are used in the high-$m_{{\mathrm {T}}}$ tail.

The multijet background is estimated from data using the same data-driven *matrix* method as used in the previous ATLAS analysis [6]. The first step of the matrix method is to calculate the fraction *f* of lepton candidates that pass the nominal lepton identification and isolation requirements (*tight*), with respect to a sample of *loose* lepton candidates in a background-enriched sample. These loosely selected candidates satisfy only a subset of the nominal criteria, which is stricter than the trigger requirements imposed. Potential contamination of prompt final-state leptons in the background-enriched sample is accounted for using MC simulation. In addition, the fraction *r* of real leptons in the sample of *loose* candidates satisfying the nominal requirements is used. This fraction is computed from MC simulation. The number of jets and photons misidentified as leptons ($N_{\mathrm T}^{\mathrm {multijet}}$) in the total number of candidates passing the signal selection ($N_{\mathrm T}$) is

1 $$\begin{aligned} N_{\mathrm T}^{\mathrm {multijet} } = f \, N_{\mathrm F} = \frac{f}{r-f} \left( \, r\, (N_{\mathrm L} + N_{\mathrm T}) - N_{\mathrm T} \right) , \end{aligned}$$

where $N_\mathrm {F}$ is the number of fake leptons and $N_\mathrm {L}$ corresponds to leptons that pass the *loose* requirements but fail the nominal requirements. As this background estimate is statistically limited at large $m_{{\mathrm {T}}}$, the expected number of events is extrapolated into the high-$m_{{\mathrm {T}}}$ region using a method similar to that for the diboson and top-quark backgrounds.

The SSM signal $W^\prime \rightarrow e \nu$ and $W^\prime \rightarrow \mu \nu$ samples were generated at LO in QCD using the Pythia 8.183 event generator and the NNPDF2.3 LO PDF set [22]. As assumed in the SSM, the couplings to fermions are equal to those of the SM *W* boson. The $W^\prime$ boson is assumed not to couple to the SM *W* and *Z* bosons and interference between the $W^\prime$ and the SM *W* boson production amplitudes is neglected. The decay $W^\prime \rightarrow \tau \nu$, where the $\tau$ subsequently decays leptonically, is not treated as part of the signal as this contribution was quantified previously and found to give a negligible contribution to the sensitivity [23]. Mass-dependent correction factors are applied to normalise the samples to the same mass-dependent NNLO pQCD calculation as used for the *W* background. Compared to the LO prediction using NNPDF2.3 LO, the corrections increase the cross-section by about 40% around a boson invariant mass of 1–2 TeV, and by about 10% at 5 TeV. Further EW corrections beyond QED FSR are not considered for the signal. The resulting cross-sections times branching ratio for $W'_{\mathrm {SSM}}$ masses of 3, 4 and 5 TeV are 15.3, 2.25 and 0.51 fb, respectively. For these $W^\prime$ masses the branching ratio to each lepton generation from Pythia is 8.2%.

The MC samples were processed through a simulation of the detector geometry and response [24] using the Geant4 [25] framework. The software used for the reconstruction is the same for both simulated and real data. The average number of pile-up interactions (additional *pp* collisions in the same or a nearby bunch crossing) observed in the data is about 23. The effect of pile-up is modelled by overlaying simulated inelastic *pp* collision events selected using very loose trigger requirements (“minimum bias”). All MC samples are reweighted so that the distribution of the number of collisions per bunch crossing matches the data. Correction factors to account for differences observed in the detector response between data and simulation are applied to the lepton trigger, reconstruction, identification [26, 27] and isolation efficiencies as well as the lepton energy/momentum resolutions and scales [27, 28].

## Event reconstruction

The analysis makes use of electrons, muons, and missing transverse momentum, whose reconstruction and identification are explained in the following.

Electrons are reconstructed from ID tracks that are matched to energy clusters in the electromagnetic calorimeter obtained using a sliding-window algorithm in the range $|\eta | < 2.47$. Candidates in the transition region between different electromagnetic calorimeter components, $1.37< |\eta | < 1.52$, are rejected. Electrons must satisfy identification criteria based on measurements of shower shapes in the calorimeter and measurements of track properties from the ID combined in a likelihood discriminant. Depending on the desired level of background rejection, *loose*, *medium* and *tight* working points are defined. Full details of the electron reconstruction, identification and selection working points can be found in Ref. [26].

Muon candidates are identified from MS tracks that match tracks in the ID, with $|\eta |<2.5$  [27]. These muons are required to pass a track quality selection based on the number of hits in the ID. They are rejected if the absolute value of the difference between their charge-to-momentum ratios measured in the ID and MS divided by the sum in quadrature of the corresponding uncertainties is large. To ensure optimal muon resolution at high $p_{\text {T}}$, additional requirements are imposed on the quality of the MS track. The track is required to have at least three hits in each of the three separate layers of MS chambers. Furthermore, to avoid $p_{\text {T}}$ mismeasurements, muons are removed if they cross either poorly aligned MS chambers, or regions in which the ID and the MS are not well aligned relative to one another.

The ID tracks associated with electron and muon candidates are required to be consistent with originating from the primary interaction vertex, which is defined as the vertex whose constituent tracks have the highest sum of $p_{\mathrm T}^2$. The transverse impact parameter with respect to the beam line, $d_0$, divided by its estimated uncertainty must satisfy $|d_0|/\sigma (d_0) < 5\ (3)$ for electrons (muons). For muons, the longitudinal impact parameter, which is the distance between the *z*-position of the point of closest approach of the muon track in the ID to the beamline and the *z*-coordinate of the primary vertex, must fulfil $|\Delta z_0| \times \sin \theta < 0.5$ mm. Both the electrons and muons are required to be isolated with respect to other particles in the event. The sum of the $p_{\text {T}}$ of tracks that fall inside an isolation cone around the lepton (excluding the track of the lepton itself) divided by the lepton $p_{\text {T}}$ has to be below a $p_{\text {T}}$-dependent threshold. The isolation cone size $\Delta R = \sqrt{(\Delta \eta )^2 + (\Delta \phi )^2}$ is defined as $10~\text {GeV}$ divided by the lepton $p_{\text {T}}$ and the cone has a maximum size of $\Delta R = 0.3$ for muons and $\Delta R = 0.2$ for electrons. For electrons, calorimeter-based isolation is also required. The sum of the calorimeter transverse energy deposits in an isolation cone of fixed size $\Delta R = 0.2$ (excluding the energy deposits of the electron itself) divided by the electron $p_{\text {T}}$ is used as the discriminating variable. The calorimeter- and track-based isolation criteria depend on $p_{\text {T}}$ and $\eta$, and are optimised for an overall efficiency of 98% (99%) for electrons (muons).

The missing transverse momentum is reconstructed as the negative vectorial sum of the calibrated momenta of electrons, muons and jets, where the electrons and muons are required to satisfy the selection criteria described above [29]. The jets used in the calculation are reconstructed in the region $|\eta | < 4.9$ from topological clusters [30] in the calorimeter using the anti-$k_t$ algorithm [31] with a radius parameter of 0.4. They are calibrated using the method described in Ref. [32] and are required to have $p_{\text {T}} ~>~20$ GeV. The computation of $E_{\text {T}}^{\text {miss}}$ also includes tracks associated with the primary vertex from activity not associated with electrons, muons or jets.

## Event selection and background estimation

Events in the muon channel were recorded by a trigger requiring that at least one muon with $p_{\text {T}} > 50$ GeV is found. These muons must be reconstructed in both the MS and the ID. In the electron channel during the 2015 (2016) data-taking period, events were recorded by a trigger requiring at least one electron with $p_{\text {T}} > 24~(60)$ GeV which satisfied the *medium* identification criteria, or at least one electron with $p_{\text {T}} > 120~(140)$ GeV which satisfied the *loose* identification criteria. The identification criteria for electrons at trigger level are similar to those used in the offline reconstruction [26].

Events recorded by the trigger are further selected by requiring that they contain exactly one lepton. In the muon channel, the magnitude of $E_{\text {T}}^{\text {miss}}$ must exceed 55 GeV and the muon has to fulfil the tight requirements for high-$p_{\text {T}}$ muons detailed in Sect. 4 and have $p_{\text {T}} > 55$ GeV. In the electron channel, the electron must satisfy the *tight* identification criteria, and the electron $p_{\text {T}}$ and the magnitude of $E_{\text {T}}^{\text {miss}}$ must both exceed 65 GeV. Events in both channels are vetoed if they contain additional leptons satisfying loosened selection criteria, namely electrons with $p_{\text {T}} > 20$ GeV satisfying the *medium* identification criteria or muons with $p_{\text {T}} > 20$ GeV passing the muon selection without the stringent requirements on the MS track quality. In addition, the transverse mass is required to exceed 110 (130) GeV in the muon (electron) channel. The acceptance times efficiency, defined as the fraction of simulated signal events that pass the event selection described above, is $50\%$ ($47\%$) for the muon channel and $81\%$ ($77\%$) for the electron channel for a $W^\prime$ mass of 2 TeV (4 TeV). The difference in lepton sensitivity results from lower muon trigger efficiency and, due to the very strict muon selection criteria applied, a lower muon identification efficiency.

**Fig. 1:**
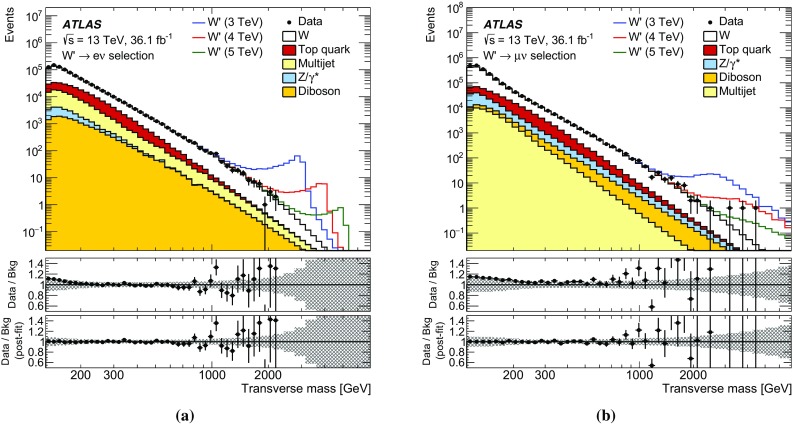
Transverse mass distributions for events satisfying all selection criteria in the **a** electron and **b** muon channels. The distributions in data are compared to the stacked sum of all expected backgrounds. As examples, expected signal distributions for three different SSM $W^\prime$ boson masses are shown on top of the SM prediction. The bin width is constant in $\log (m_{\mathrm {T}})$. The middle panels show the ratios of the data to the expected background, with vertical bars representing both data and MC statistical uncertainties. The lower panels show the ratios of the data to the adjusted expected background (“post-fit”) that results from the statistical analysis. The bands in the ratio plots indicate the sum in quadrature of the systematic uncertainties discussed in Sect. 6, including the uncertainty in the integrated luminosity

**Table 1:** The numbers of expected events from the total SM background and SSM $W^\prime$ signal and the numbers of observed events in data in the electron (top) and muon (bottom) channels in bins of $m_{{\mathrm {T}}}$. The uncertainties given are the combined statistical and systematic uncertainties. The systematic uncertainty includes all systematic uncertainties except the one from the integrated luminosity (3.2%)

Electron channel							
$m_\mathrm {T}$ (GeV)	130–200	200–400	400–600	600–1000	1000–2000	2000–3000	3000–7000
Total SM	620,000 ± 70,000	168,000 ± 10,000	9700 ± 500	2010 ± 140	232 ± 24	5.9 ± 1.4	0.4 ± 0.4
$W^\prime$ (2 TeV)	24.3 ± 0.9	126 ± 3	199 ± 5	614 ± 14	3280 ± 50	330 ± 70	0.85 ± 0.04
$W^\prime$ (3 TeV)	3.83 ± 0.08	14.2 ± 0.2	16.1 ± 0.4	35.7 ± 0.4	122 ± 2	229 ± 4	24 ± 5
$W^\prime$ (4 TeV)	1.18 ± 0.02	4.06 ± 0.03	3.58 ± 0.03	5.92 ± 0.03	12.1 ± 0.1	13.5 ± 0.2	23.3 ± 0.2
$W^\prime$ (5 TeV)	0.476 ± 0.008	1.62 ± 0.01	1.35 ± 0.01	1.95 ± 0.01	2.64 ± 0.01	1.56 ± 0.01	3.72 ± 0.02
Data	671,128	169,338	9551	1931	246	4	0

Muon channel							
$m_\mathrm {T}$ (GeV)	110–200	200–400	400–600	600–1000	1000–2000	2000–3000	3000–7000
Total SM	1,640,000 ± 200,000	122,000 ± 8000	6460 ± 330	1320 ± 90	150 ± 13	4.7 ± 0.6	0.63 ± 0.13
$W^\prime$ (2 TeV)	25.0 ± 1.5	102 ± 6	143 ± 9	420 ± 22	1720 ± 90	369 ± 28	17 ± 4
$W^\prime$ (3 TeV)	3.98 ± 0.12	10.3 ± 0.3	10.7 ± 0.5	26.3 ± 1.5	84 ± 5	98 ± 6	39.3 ± 3.4
$W^\prime$ (4 TeV)	1.20 ± 0.03	2.80 ± 0.07	2.36 ± 0.09	4.07 ± 0.19	8.1 ± 0.5	8.8 ± 0.6	11.1 ± 0.9
$W^\prime$ (5 TeV)	0.485 ± 0.012	1.12 ± 0.03	0.88 ± 0.03	1.27 ± 0.05	1.7 ± 0.1	0.99 ± 0.07	1.7 ± 0.1
Data	1,862,326	128,155	6772	1392	177	3	3

The expected number of background events is calculated as the sum of the data-driven and simulated background estimates described in Sect. 3. Figure 1 displays the $m_{{\mathrm {T}}}$ distribution in the electron and muon channels. The expected and observed number of events for some wider $m_{{\mathrm {T}}}$ ranges are shown also in Table 1. For all values of $m_{{\mathrm {T}}}$, the background is dominated by $W \rightarrow \ell \nu$ production, which constitutes about 85% of the total background at $m_{{\mathrm {T}}}$ $>1$ TeV. As examples, Fig. 1 also shows the expected signal distributions for three assumed $W'_{\mathrm {SSM}}$ boson masses on top of the SM prediction. The effect of the momentum resolution is clearly visible when comparing the shapes of the three reconstructed $W'_{\mathrm {SSM}}$ signals in the electron and muon channels. The middle panels of Fig. 1 show the ratio of the data to the SM predictions. The data are systematically above the predicted background at low $m_{{\mathrm {T}}}$, but still within the total systematic uncertainty, which is dominated by the $E_{\text {T}}^{\text {miss}}$ -related systematic uncertainties in this region. The bottom panels of Fig. 1 show the ratio of the data to the adjusted background that results from a common fit to the electron and muon channels within the statistical analysis described in Sect. 7. This ratio agrees well with unity.

## Systematic uncertainties

**Table 2:** Systematic uncertainties in the expected number of events as estimated for the total background and for signal with a $W'_{\mathrm {SSM}}$ mass of 2 (4) TeV. The uncertainty is estimated with the binning shown in Fig. 1 at $m_{{\mathrm {T}}}$ $=$ 2 (4) TeV for the background and in a three-bin window around $m_{{\mathrm {T}}}$ $=$ 2 (4) TeV for the signal. Uncertainties that are not applicable are denoted “n/a”, and “negl.” means that the uncertainty is not included in the statistical analysis. Sources of uncertainties not included in the table are neglected in the statistical analysis.

Source	Electron channel		Muon channel	
	Background	Signal	Background	Signal
Trigger	negl. (negl.)	negl. (negl.)	${2}$% (${2}$%)	${2}$% (${2}$%)
Lepton reconstruction and identification	negl. (negl.)	negl. (negl.)	${5}$% (${6}$%)	${5}$% (${7}$%)
Lepton momentum scale and resolution	${3}$% (${3}$%)	${4}$% (${3}$%)	${3}$% (${9}$%)	${1}$% (${1}$%)
$E_{\text {T}}^{\text {miss}}$ resolution and scale	${<0.5}$% (${<0.5}$%)	${<0.5}$% (${<0.5}$%)	${<0.5}$% (${1}$%)	${1}$% (${1}$%)
Jet energy resolution	${<0.5}$% (${<0.5}$%)	${<0.5}$% (${<0.5}$%)	${<0.5}$% (${<0.5}$%)	${<0.5}$% (${<0.5}$%)
Pile-up	${1}$% (${<0.5}$%)	${1}$% (${<0.5}$%)	${<0.5}$% (${1}$%)	${1}$% (${<0.5}$%)
Multijet background	${7}$% (${70}$%)	n/a (n/a)	${1}$% (${1}$%)	n/a (n/a)
Top extrapolation	${1}$% (${1}$%)	n/a (n/a)	${4}$% (${8}$%)	n/a (n/a)
Diboson extrapolation	${4}$% (${20}$%)	n/a (n/a)	${4}$% (${10}$%)	n/a (n/a)
PDF choice for DY	${1}$% (${13}$%)	n/a (n/a)	${<0.5}$% (${1}$%)	n/a (n/a)
PDF variation for DY	${8}$% (${15}$%)	n/a (n/a)	${7}$% (${11}$%)	n/a (n/a)
EW corrections for DY	${4}$% (${7}$%)	n/a (n/a)	${4}$% (${5}$%)	n/a (n/a)
Luminosity	${3}$% (${3}$%)	${3}$% (${3}$%)	${3}$% (${3}$%)	${3}$% (${3}$%)
Total	${13}$% (${76}$%)	${5}$% (${5}$%)	${12}$% (${21}$%)	${6}$% (${8}$%)

The systematic uncertainties arise from experimental and theoretical sources. They are summarised in Table 2 and described in the following subsections.

### Uncertainties from the reconstruction of electrons, muons, and $E_{\text {T}}^{\text {miss}}$

Experimental systematic uncertainties arise from the trigger, reconstruction, identification and isolation efficiencies for leptons [26, 27], and the calculation of the missing transverse momentum [29]. They include also the effects of the energy and momentum scale and resolution uncertainties [27, 28, 32].

The electron and muon offline reconstruction, identification and isolation efficiencies, and their respective uncertainties, are assessed up to $p_{\text {T}} \approx 100$ GeV using leptonic decays of *Z* boson candidates found in data. The ratio of the efficiency measured in data to that of the MC simulation is then used to correct the MC prediction [26, 27]. For higher-$p_{\text {T}}$ electrons, an additional uncertainty of 1.5% is estimated for the *tight* identification working point. This uncertainty is based on the differences observed in the electron shower shapes in the EM calorimeters between data and MC simulation around the $Z \rightarrow ee$ mass peak, which are propagated to the high-$E_{\text {T}}$ electron sample. For the isolation efficiencies, an uncertainty of 2 and 5% is estimated for $150< p_{\text {T}} < 500$ GeV and above 500 GeV, respectively, using $Z/\gamma ^*$ candidates in data. For the identification of high-$p_{\text {T}}$ muons, the uncertainty is determined conservatively from simulation studies and amounts to 2–3% per TeV. For the isolation criterion, the uncertainty associated with the extrapolation to high-$p_{\text {T}}$ muons is estimated to be 1%. Systematic uncertainties related to the electron trigger are negligible. For the muon trigger the systematic uncertainty is estimated using the same methodology as in Ref. [33], which results in an overall uncertainty of about 2%.

The main systematic uncertainties in $E_{\text {T}}^{\text {miss}}$ arise from the jet energy resolution uncertainties [32] and the contribution from tracks originating from the primary vertex and arising from activity not associated with electrons, muons or jets [29]. The uncertainties due to the jet energy and $E_{\text {T}}^{\text {miss}}$ resolutions are small at large $m_{{\mathrm {T}}}$, while they are the dominant contributions to the total uncertainty at small $m_{{\mathrm {T}}}$. The jet energy scale uncertainties are found to be negligible.

### Theoretical uncertainties

Theoretical uncertainties are related to the production cross-sections estimated from MC simulation. The effects when propagated to the total background estimate are significant for *W* and $Z/\gamma ^*$ production, but negligible for top-quark and diboson production. No theoretical uncertainties are considered for the $W^\prime$ boson signal in the statistical analysis.

Theoretical uncertainties in the *W* and $Z/\gamma ^*$ background prediction arise from the PDF uncertainties, the value of the strong coupling constant $\alpha _{\mathrm {s}}$, and higher-order corrections. The dominant effect comes from the PDF uncertainty, which is obtained from the 90% CL CT14NNLO PDF uncertainty set using VRAP to calculate the NNLO cross-section as a function of the boson mass. Rather than using the original 28 CT14 uncertainty eigenvectors, a re-diagonalised set of seven PDF eigenvectors, as provided by the authors of the CT14 PDF using MP4LHC [34, 35], is used. The cross-section variation associated with each of these eigenvectors has a characteristic mass dependence and the sum in quadrature of these eigenvector variations matches the original CT14NNLO uncertainty envelope well. This sum is shown as “PDF variation” in Table 2. An additional uncertainty is derived to account for the choice of the nominal PDF set used. The central values of the CT14NNLO PDF set are compared to the MMHT2014 [36] and NNPDF3.0 [37] PDF sets. A comparison between these PDF sets shows that the central value for NNPDF3.0 falls outside the “PDF variation” uncertainty at large $m_{{\mathrm {T}}}$. Thus, an envelope of the “PDF variation” and the NNPDF3.0 central value is formed, where the former is subtracted in quadrature from this envelope, and the remaining part, which is non-zero only when the NNPDF3.0 central value is outside the “PDF variation” uncertainty, is quoted as “PDF choice”. The PDF uncertainties are the same at the generator level for the electron and muon channels, but result in different uncertainties at reconstruction level. The uncertainty is larger in the electron channel due to the better energy resolution: there is less migration of events with low generator-level invariant mass, where the PDF uncertainty is smaller, into the high-$m_{{\mathrm {T}}}$ region in this channel.

Uncertainties in the electroweak corrections are determined as the difference between the additive approach ($1+\delta _{\mathrm {EW}} + \delta _{\mathrm {QCD}}$) and a factorised approach ($(1+\delta _{\mathrm {EW}}) \times (1+\delta _{\mathrm {QCD}})$) for the EW corrections in the combination of higher-order EW ($\delta _{\mathrm {EW}}$) and QCD ($\delta _{\mathrm {QCD}}$) effects. Uncertainties due to higher-order QCD corrections on the $Z/\gamma ^*$ process are estimated by varying the renormalisation and factorisation scales simultaneously up or down by a factor of two. The uncertainty due to $\alpha _{\mathrm {s}}$ is assessed by changing the value of $\alpha _{\mathrm {s}}$ by as much as 0.003 from the nominal value $\alpha _{\mathrm {s}}(m_Z) = 0.118$ used by the CT14NNLO PDF set. The uncertainties from the scales and $\alpha _{\mathrm {s}}$ are both found to be negligible.

Theoretical uncertainties are also considered for the top-quark and diboson backgrounds. An uncertainty in the $t\bar{t}$ cross-section of $^{+20}_{-29}$ pb arises from the independent variation of the factorisation and renormalisation scales, while an uncertainty of $\pm 35$ pb is associated with variations in the PDF and $\alpha _{\mathrm {s}}$, following the PDF4LHC prescription (see Ref. [38] and references therein) with the MSTW2008 68% CL NNLO [39], CT10 NNLO [40] and NNPDF2.3 NNLO [22] PDF sets. As this background constitutes only a small fraction of the overall background, these normalisation uncertainties are negligible. Furthermore, the modelling of the top-quark background is found to be adequate in a data control region defined by requiring the presence of an additional muon (electron) in events passing the electron (muon) selection. For the diboson background, the theoretical normalisation uncertainty is conservatively taken to be 30%, and this has a negligible effect due to the small contribution of this background.

### Background modelling uncertainties

The dominant systematic uncertainties in the multijet, top-quark and diboson backgrounds at high $m_{{\mathrm {T}}}$ are due to the extrapolations. These uncertainties are evaluated by varying both the functional form of the fit functions and the fit range as detailed in Sect. 3. The envelope of all variations is assigned for the uncertainty. This results in the largest source of background-related systematic uncertainty at large $m_{{\mathrm {T}}}$ values in this analysis.

The multijet background uncertainty in the electron (muon) channel includes a 15% (100%) normalisation uncertainty. This uncertainty is dominated by the dependence of the factor *f* (see Sect. 3) on the selection requirements used for the background-enriched sample definition.

For the $m_{{\mathrm {T}}}$ region below 700–800 GeV, for which there are not many more MC events than data events, the MC statistical uncertainty is accounted for in the analysis.

The modelling of the pile-up especially affects the calculation of $E_{\text {T}}^{\text {miss}}$ . A pile-up modelling uncertainty is estimated by varying the distribution of pile-up events in the reweighting of the MC, as detailed in Sect. 3, to cover the uncertainty on the ratio between the predicted and measured inelastic cross-sections [41].

### Luminosity

The uncertainty in the combined 2015 and 2016 integrated luminosity is 3.2%. Following a methodology similar to that detailed in Ref. [42], it is derived from a preliminary calibration of the luminosity scale using *x*–*y* beam-separation scans performed in August 2015 and May 2016.

## Results

For the statistical analysis of the results presented in this section, the same methodology is applied as in the previous ATLAS $W^\prime$ search [6] and is described briefly here. The compatibility between the data and the predicted background is evaluated with a profile-likelihood ratio test quantifying the probability that the background fluctuates to give a signal-like excess equal to or larger than what is observed. The likelihood functions in the ratio are products of Poisson probabilities over all bins in the transverse mass distribution (as shown in Fig. 1) and log-normal constraints for the variations in signal and background yields associated with systematic uncertainties. In the denominator of the likelihood ratio, the likelihood function is maximised assuming the presence of a signal above the expected background, and in the numerator assuming the background-only hypothesis. To model the signal, $W'_{\mathrm {SSM}}$ templates binned in $m_{{\mathrm {T}}}$ are used for a series of $W'_{\mathrm {SSM}}$ masses in the search range $150~\text {GeV}\ \le$ $m_{W'}$ $\le 6000~\text {GeV}$. Figure 1 displays a few examples of these templates. No significant excesses are observed in the data. The most significant excess is at $m_{W^\prime } = 350$ GeV in the electron channel, with a local significance of $2.0 \sigma$. In the muon channel, the most significant excess is at high mass, with a maximum local significance of $1.8 \sigma$ at $m_{W^\prime } \approx 5$ TeV. These excesses correspond to a global significance of $0.1 \sigma$ in each channel when the look-elsewhere effect [43] is taken into account.

Based on the above findings, upper limits on the cross-section for producing a $W'_{\mathrm {SSM}}$ boson times its branching ratio to only one lepton generation ($\sigma \times {\textit{BR}}$) are computed at the 95% CL as a function of the $W'_{\mathrm {SSM}}$ boson mass. The limits are calculated in a Bayesian analysis [44] with a uniform positive prior probability distribution for $\sigma \times {\textit{BR}}$. The observed upper limits are extracted by comparing data to the expected background and signal using $W'_{\mathrm {SSM}}$ templates for the same range of signal masses as for the profile-likelihood ratio test. The expected limits are derived from pseudo-experiments obtained from the estimated background distributions. The median of the distribution of the limits from the pseudo-experiments is taken as the expected limit, and $1 \sigma$ and $2 \sigma$ bands are defined as the ranges containing respectively 68 and 95% of the limits obtained with the pseudo-experiments.

**Fig. 2:**
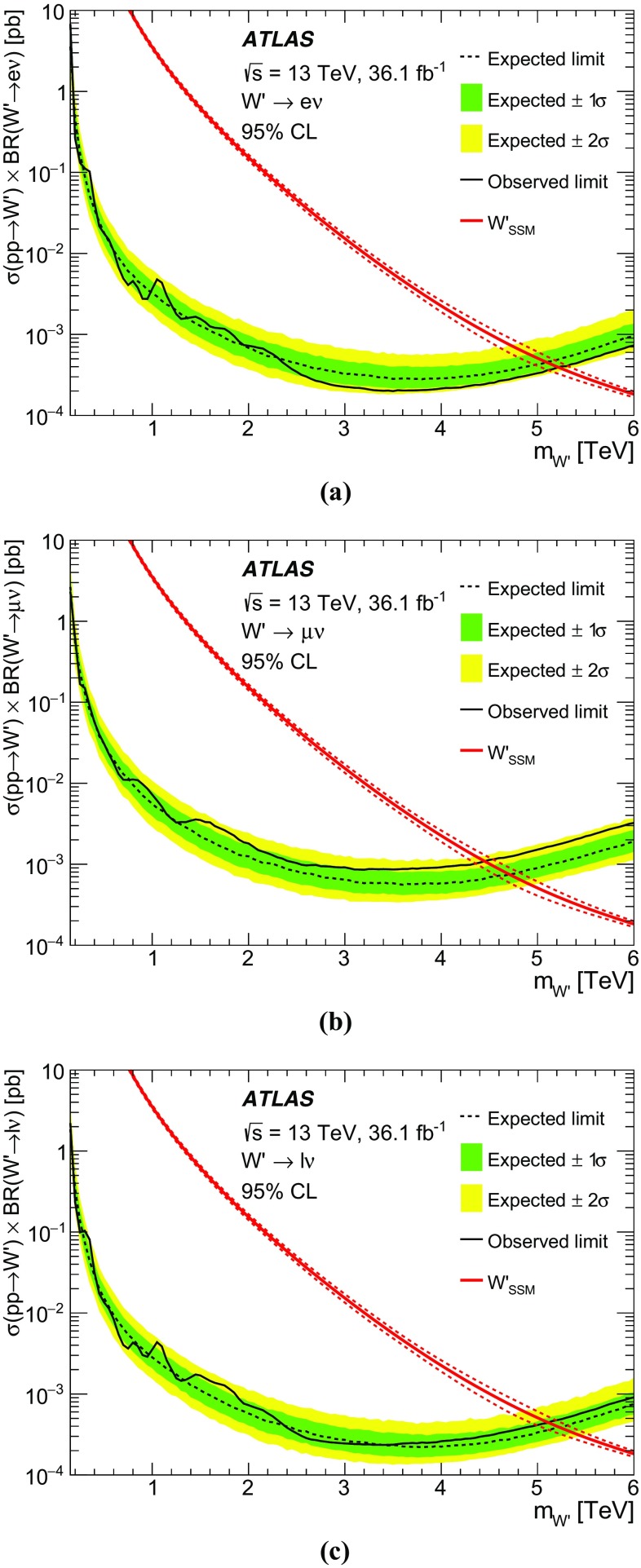
Observed (solid black line) and expected (dashed black line) upper limits on cross-section times branching ratio ($\sigma \times {\textit{BR}}$) as a function of the SSM $W^\prime$ boson mass in the **a** electron, **b** muon and **c** combined electron and muon channels. The $1\sigma$ (green) and $2\sigma$ (yellow) expected limit bands are also shown. The predicted $\sigma \times {\textit{BR}}$ for SSM $W^\prime$ production is shown as a red solid line. For illustration the uncertainties in $\sigma \times {\textit{BR}}$ from the PDF, $\alpha _{\mathrm {s}}$ and the renormalisation and factorisation scales are also shown as red-dashed lines

The 95% CL upper limits on $\sigma \times {\textit{BR}}$ as a function of the $W'_{\mathrm {SSM}}$ mass are shown in Fig. 2 separately for the electron and muon channels and for the combination of the two channels. The theoretical uncertainties and the uncertainties in $E_{\text {T}}^{\text {miss}}$ , jet energy resolution and luminosity are treated as correlated between the channels. The expected upper limit on $\sigma \times {\textit{BR}}$ is stronger in the electron channel. This results from the larger acceptance times efficiency and the better momentum resolution (see Sect. 5). Figure 2 also shows the predicted $\sigma \times {\textit{BR}}$ for the $W'_{\mathrm {SSM}}$ boson as a function of its mass as well as the uncertainties from the PDF, $\alpha _{\mathrm {s}}$ and the factorisation and renormalisation scales derived using the same prescription as used for the *W* boson production. The observed (expected) lower mass limit for a $W'_{\mathrm {SSM}}$ boson, as summarised in Table 3, is 5.1 (5.2) TeV for the combination of the electron and muon channels. This corresponds to an improvement of approximately 1 TeV in mass reach compared to the previous ATLAS analysis [6], which was based on a subset of the data used in this analysis.

**Table 3:** Expected and observed 95% CL lower limit on the $W'_{\mathrm {SSM}}$ mass in the electron and muon channels and their combination

Decay	$m_{W'}$ lower limit (TeV)	
	Expected	Observed
$W'\rightarrow e \nu$	5.1	5.2
$W'\rightarrow \mu \nu$	4.7	4.5
$W'\rightarrow \ell \nu$	5.2	5.1

## Conclusion

The results of a search for a new heavy gauge boson decaying to final states with a high-$p_{\text {T}}$ electron or muon and large missing transverse momentum are reported. The analysis uses 36.1 $\text{ fb }^{-1}$ of $\sqrt{s} = 13$ TeV *pp* collision data recorded by the ATLAS detector at the Large Hadron Collider in 2015 and 2016. Examining the transverse mass spectrum, no significant excess above the expected Standard Model background is observed. Exclusion limits at 95% CL are placed on the mass of benchmark Sequential Standard Model $W^\prime$ bosons. Masses for $W'_{\mathrm {SSM}}$ bosons up to 5.1 TeV are excluded by the combination of the electron and muon channels. This exceeds the previous limit from ATLAS, derived from a similar analysis based on 3.2 $\text{ fb }^{-1}$ of $\sqrt{s} = 13$ TeV data, by 1 TeV.

## Footnotes

1 ATLAS uses a right-handed coordinate system with its origin at the nominal interaction point (IP) in the centre of the detector and the *z*-axis along the beam pipe. The *x*-axis points from the IP to the centre of the LHC ring, and the *y*-axis points upward. Cylindrical coordinates $(r,\phi )$ are used in the transverse plane, $\phi$ being the azimuthal angle around the *z*-axis. The pseudorapidity is defined in terms of the polar angle $\theta$ as $\eta =-\ln \tan (\theta /2)$. Transverse momentum ($p_{\text {T}}$) is defined relative to the beam axis and is calculated as $p_{\text {T}} = p \sin (\theta )$ where *p* is the momentum.
